# The prognostic significance of JAML and its role in remodeling the immune microenvironment via the cGAS-STING pathway in endometrial cancer

**DOI:** 10.3389/fimmu.2026.1738596

**Published:** 2026-01-29

**Authors:** Chenfan Tian, Yuanyang Yao, Yuan Tu, Jiaxin Yu, Chunxia Gong, Xiuling Shi, Hangkun Yu, Peng Jiang

**Affiliations:** 1 Chongqing Key Laboratory of Maternal and Fetal Medicine, Department of Obstetrics and Gynecology, The First Affiliated Hospital of Chongqing Medical University, Chongqing, China; 2 Department of Obstetrics and Gynecology, Peking University People's Hospital, Beijing, China; 3 Department of Obstetrics and Gynecology, Women and Children’s Hospital of Chongqing Medical University, Chongqing, China

**Keywords:** chemosensitivity, endometrial carcinoma, immunotherapy, JAML, nomogram model, prognosis

## Abstract

**Background:**

Endometrial cancer (EC) is a major gynecological malignancy with a poor prognosis in the advanced stages. Junctional adhesion molecule-like protein (JAML) is gaining attention in cancer biology; however, its role in EC remains unclear.

**Methods:**

This study integrated multi-omics data (TCGA, pan-cancer analysis and single-cell transcriptomics), dual independent clinical cohorts (TCGA dataset and an institutional cohort), and *in vitro*/*in vivo* experimental models to systematically analyze JAML expression patterns, clinical relevance, and tumor-progression regulatory mechanisms in EC. Experimental approaches included: immunohistochemistry (IHC), qRT-PCR, and Western blotting for expression validation; shRNA knockdown, Transwell/scratch assays, CCK-8 assays, and conditioned medium co-culture models for functional analysis; bioinformatics tools (ESTIMATE, TIMER, MCP-counter) and flow cytometry for immune microenvironment evaluation; drug sensitivity analysis (Genomics of Drug Sensitivity in Cancer (GDSC)/Tumor Immune Dysfunction and Exclusion (TIDE) databases) and prognostic modeling (Cox regression/nomogram) for clinical application assessment.

**Results:**

In EC, JAML expression was significantly downregulated and correlated with poor prognosis. JAML knockdown promoted cancer cell proliferation, migration, invasion, and epithelial–mesenchymal transition (EMT) (all P < 0.01), accompanied by reduced stimulator of interferon genes (STING) phosphorylation (P < 0.01). Low JAML expression correlated with decreased M1 (CD86^+^, P < 0.01) and increased M2 (CD206^+^, P < 0.01) macrophage infiltration. It was also linked to reduced cisplatin/paclitaxel sensitivity (P < 0.05) and lower immunotherapy response (29.04% vs. 45.05%, P = 0.015). STING agonist cyclic GMP–AMP (cGAMP) partially restored M1 infiltration and chemosensitivity after JAML knockdown *in vitro*. A nomogram incorporating JAML and clinicopathological parameters showed improved predictive performance (AUC 0.885; cutoff 0.882; sensitivity 0.818, specificity 0.820), with calibration curves confirming good agreement between predicted and observed recurrence.

**Conclusions:**

These findings suggest that JAML deficiency may suppress cyclic GMP-AMP synthase (cGAS)-STING pathway activation, potentially contributing to M1/M2 macrophage polarization imbalance and facilitating EC progression. Clinically, JAML expression shows promise as a potential biomarker for prognostic stratification and treatment response prediction (chemotherapy/immunotherapy), providing insights for developing precision immunochemotherapy strategies in EC.

## 1 Introduction

Endometrial cancer (EC) ranks as the sixth most frequent malignancy in women, with over 420,000 new cases annually worldwide (1). Its global incidence has increased by approximately 21% over the past two decades, with population ageing and the rising prevalence of obesity being key contributing factors (2). Surgical intervention represents the primary treatment for early-stage disease, with adjuvant therapy guided by pathological risk assessment (3). However, approximately 15% of patients are diagnosed with advanced-stage disease. Among these advanced-stage patients, prognosis varies substantially, with reported 5-year overall survival (OS) rates declining from 45–50% for International Federation of Gynecology and Obstetrics (FIGO) stage III to 13–17% for stage IV (4). The management of recurrent or metastatic EC remains a clinical challenge, where chemotherapy has historically been the mainstay despite its reduced efficacy in later treatment lines (5). While molecular classification systems such as The Cancer Genome Atlas (TCGA) and the advent of immunotherapy have transformed the therapeutic landscape, clinical benefits appear largely restricted to specific molecular subtypes, with primary and acquired resistance representing significant barriers (6). Therefore, identifying new molecular markers that can predict treatment response and prognosis is of great significance for advancing precision medicine in EC.

Junctional Adhesion Molecule-Like (JAML), a key member of the JAM family, is implicated in immune regulation (7). It facilitates inflammatory responses by mediating intercellular adhesion and directional migration, and is expressed on immune cells including neutrophils, monocytes, and T lymphocytes (8–11). JAML exhibits diverse functions in cancer biology. In immune cells like γδ T cells and CD8^+^ T cells, it has been reported to function as a co-stimulatory receptor that binds CXADR to enhance anti-tumor immunity, correlating with better patient outcomes (12). This apparent anti-tumor role contrasts with findings in specific cancer types, suggesting potential context-dependent functions. Separately, studies in lung and gastric adenocarcinomas have shown that cancer cell-intrinsic JAML can promote malignant behaviors, including proliferation, migration, and immune evasion, via activation of the Wnt/β-catenin and p38 pathways (13, 14). This divergence in reported effects may reflect tissue-specific or microenvironmental influences on JAML function. Although JAML’s roles in several cancers are emerging, its expression, prognostic significance, and functional mechanisms in EC remain unclear.

This study aims to elucidate the prognostic significance and immunoregulatory mechanisms of JAML in EC. We hypothesize that JAML deficiency drives EC progression by suppressing cyclic GMP-AMP synthase-stimulator of interferon genes (cGAS-STING) pathway activation, thereby impairing anti-tumor immunity and inducing chemotherapy resistance. To test this hypothesis and leverage the established prognostic value of JAML expression, we developed an integrated prognostic model combining JAML levels with clinicopathological features. This model serves to identify high-risk patients and guide individualized therapeutic management.

## 2 Materials and methods

### 2.1 Bioinformatics analysis of JAML expression patterns

Transcriptomic data from TCGA Uterine Corpus Endometrial Carcinoma (UCEC) project were integrated with normal endometrial tissue data from the Genotype-Tissue Expression (GTEx) v8 database. JAML expression, quantified as log_2_ (transcripts per million [TPM] + 1), was compared between tumor and normal tissues using Wilcoxon rank-sum tests, and across FIGO stages and histological grades using Kruskal-Wallis tests. Associations with molecular subtypes were analyzed via the TISIDB portal. Protein-level validation was performed using immunohistochemical images sourced from The Human Protein Atlas (HPA) (15). Detailed information regarding the bioinformatics tools, including their websites, can be found in **Supplementary Table 1**.

### 2.2 Survival, functional enrichment and immune analysis

OS and recurrence-free survival (RFS) were analyzed using the Kaplan-Meier method, with patients stratified by the optimal JAML expression cutoff determined from the TCGA-UCEC cohort, and between-group differences assessed by the log-rank test (16). Protein-protein interaction (PPI) networks were constructed using GeneMANIA (prioritizing physical interactions and co-expression) and STRING (generated from the top 1000 JAML-co-expressed genes in the EC cohort, with a maximum of 20 interactors) (17, 18). To systematically interpret the functional implications, we performed both Gene Set Enrichment Analysis (GSEA) with significance thresholds of nominal P < 0.05 and FDR q < 0.25 (19), and functional enrichment analysis of Gene Ontology (GO) terms and Kyoto Encyclopedia of Genes and Genomes (KEGG) pathways using Metascape (20).

For immune landscape characterization, transcriptomic data from TCGA-UCEC were analyzed using the ESTIMATE algorithm to compute immune, stromal, and combined ESTIMATE scores. Tumor purity was inferred from the ESTIMATE score using the published formula: Purity = cos(0.6049872018 + 0.000146788 × ESTIMATE score) (21). To further assess the abundance of immune cell infiltration in heterogeneous endometrial cancer tissues, the Microenvironment Cell Populations-counter (MCP-counter) algorithm was employed (15). These metrics were subsequently compared between JAML-high and JAML-low subgroups. In addition, the TISIDB portal was employed to investigate correlations between JAML expression and a comprehensive set of immune modulators, including immunostimulators, Major Histocompatibility Complex (MHC) molecules, tumor-infiltrating lymphocytes (TILs), and chemokines (22).

### 2.3 Immunotherapy and chemotherapy response assessment

Immunotherapy response was predicted using the Tumor Immune Dysfunction and Exclusion (TIDE) framework, which evaluates tumor immune evasion by integrating cytotoxic T lymphocyte dysfunction and exclusion metrics to predict immune checkpoint blockade efficacy (23, 24). Higher TIDE scores indicate poorer response. Chemosensitivity was assessed by analyzing correlations between JAML expression and half-maximal inhibitory concentrations (IC_50_) of cisplatin and paclitaxel using the Genomics of Drug Sensitivity in Cancer (GDSC) database (25).

### 2.4 Patient cohort and sample processing

This retrospective study enrolled 722 patients with stage I-III EC, staged according to the FIGO 2009 criteria (26), who underwent primary surgery between January 2018 and January 2022. These patients were assigned to a training cohort (n=483) from the First Affiliated Hospital and a validation cohort (n=239) from the Women and Children’s Hospital of Chongqing Medical University (WCHCQMU). Exclusion criteria covered several scenarios: undergoing non-standard surgery, having a sarcoma pathological type, presenting with metastatic disease upon initial diagnosis, harboring synchronous malignancies, receiving neoadjuvant therapy, or cases where follow-up data were unavailable. Clinical data were abstracted from medical records: age, body-mass index (BMI), histologic subtype, grade, depth of myometrial and cervical invasion, lymphovascular space invasion (LVSI), pre-operative serum CA125, and any adjuvant therapy administered postoperatively.

Pathology center archives provided postoperative tissue specimens, which were fashioned into paraffin sections for immunohistochemical analysis. Moreover, 20 paired samples of fresh EC and adjacent normal endometrium were gathered for further validation via qRT-PCR and western blotting. The protocols for postoperative adjuvant therapy, patient follow-up, and the definition of recurrence were consistent with those described in a previous publication (27). The study was conducted in accordance with the Declaration of Helsinki and was approved by the Institutional Review Boards of all participating hospitals (Approval Nos.: 2025-283–01 and 2023-002).

### 2.5 Immunotherapy cohort and efficacy evaluation

To validate the predictive value of JAML expression for the response to an immune checkpoint inhibitor−based treatment regimen, we established a validation cohort. This study enrolled 39 patients with advanced (FIGO stage III-IV) EC who were treated at the First Affiliated Hospital of Chongqing Medical University (FAHCQMU) between January 2021 and December 2022. Key inclusion criteria were: histologically confirmed diagnosis, age ≥18 years, and receipt of at least one cycle of neoadjuvant chemotherapy (TP regimen) combined with the PD-1 inhibitor camrelizumab. Patients were excluded for: (1) active autoimmune diseases; (2) lack of archived tumor tissue sufficient for biomarker analysis; (3) incomplete follow-up data; or (4) other concurrent active primary malignancies (excluding metastatic foci of EC).

Treatment efficacy in this cohort was evaluated by two independent radiologists according to the Response Evaluation Criteria in Solid Tumors (RECIST) version 1.1 (28), with a third radiologist adjudicating discrepancies. Radiographic assessments were repeated every 6–8 weeks. The primary endpoints were Objective Response Rate (ORR), defined as the proportion of patients with a confirmed complete or partial response (requiring maintenance for ≥4 weeks), and Progression-Free Survival (PFS), defined as the time from treatment initiation to radiological progression or death from any cause. Progression was defined as a ≥20% increase in the sum of diameters of target lesions (with an absolute increase of ≥5 mm) compared to the nadir, and/or the appearance of new lesions, and/or unequivocal progression of non-target lesions.

### 2.6 Immunohistochemistry

IHC was conducted following established protocols (27) with the following primary antibodies: JAML (Proteintech, 21302-1-AP; 1:200), p-STING (Thermo Fisher, PA5-105674; 1:100), CD86 (Abcam, ab243887; 1:500), and CD206 (Proteintech, 18704-1-AP; 1:4000). Staining was assessed in five randomly selected high-power fields (400×) within the most active tumor regions. For JAML and p-STING, a semiquantitative scoring system (range 1–4) was applied, calculated as the product of positive cell percentage and staining intensity. Samples with scores ≥2.5 (median cutoff) were classified as high-expression. Assessment of CD86^+^/CD206^+^ macrophages and p53 status followed previously published criteria (29). All slides were evaluated independently by two pathologists in a blinded fashion, with inter-observer reliability assessed by Cohen’s κ (for dichotomized JAML) and intraclass correlation coefficients (ICCs) (for continuous scores of p−STING and quantitative counts of CD86^+^/CD206^+^), as reported in **Supplementary Tables 2** and **3**. Discordant cases were resolved through consensus review.

### 2.7 Western blotting

Western blot analysis was performed following standard procedures (30). Proteins were extracted using RIPA lysis buffer (Beyotime, China) supplemented with PMSF and phosphatase inhibitors, with concentrations determined by BCA assay (Beyotime, China). After denaturation, samples were separated by 10% SDS-PAGE and transferred to PVDF membranes. Blocking was carried out with 5% skim milk in TBST, followed by incubation with primary antibodies: JAML (Proteintech, 21302-1-AP; 1:2000), Ki-67 (Santa Cruz Biotechnology, sc-23900; 1:500), N-cadherin (Proteintech, 82968-1-RR; 1:10,000), E-cadherin (Proteintech, 20874-1-AP; 1:50,000), vimentin (Proteintech, 80232-1-RR; 1:20,000), cGAS (Abmart, PQA3430; 1:1000), STING (Abmart, TD12090; 1:1000), p-STING (Thermo Fisher, PA5-105674; 1:1000), TBK1 (Proteintech, 28397-1-AP; 1:1000), p-TBK1 (GenuIN, 61294; 1:2500), IRF3 (GenuIN, 61219; 1:2500), p-IRF3 (Proteintech, 29528-1-AP; 1:2000) and GAPDH (Proteintech, 60004-1-Ig; 1:50,000). Membranes were then incubated with HRP-conjugated secondary antibodies (Proteintech, SA00001-2, SA00001-1; 1:5000) and visualized using WesternBright™ ECL substrate (Advansta, USA) on a ChemiDoc Touch system (Bio-Rad, USA).

### 2.8 qRT-PCR

Total RNA was extracted using an RNA Rapid Extraction Kit (Yisha, China), with concentration and purity verified by spectrophotometry. cDNA was synthesized with RT-Master Mix for qPCR II (MedChemExpress, USA). qRT-PCR was performed on a Bio-Rad CFX96 real-time PCR system using SYBR Green PCR Kit (MedChemExpress, USA) (29). β-actin served as the endogenous control. Primer sequences were: JAML: Forward: 5′-AGAGCACGCCAAGGACGAATA-3′, Reverse: 5′-GGAGCAGGAGAGAGCCATCAT-3′; β-actin: Forward: 5′-AGAAATCTGGCACCACACCT-3′, Reverse: 5′-GATAGCACAGCCTGGATAGCA-3′; STING: Forward: 5’-CCAGAGCACACTCTCCGGTA-3′, STING Reverse: 5’-CGCATTTGGGAGGGAGTAGTA-3′.

### 2.9 Cell culture

The human endometrial adenocarcinoma cell lines Ishikawa and HEC-1A, along with the human monocytic cell line THP-1, were obtained from the Cell Bank of the Chinese Academy of Sciences. Ishikawa and HEC-1A cells were maintained in high-glucose DMEM (Gibco, USA) supplemented with 10% fetal bovine serum (FBS; NSERA, Uruguay) and 1% penicillin-streptomycin (Beyotime, China) (31). THP-1 cells were cultured in RPMI 1640 medium (Gibco, USA) containing 10% FBS (32). All cell lines were incubated at 37 °C in a humidified atmosphere with 5% CO_2_.

### 2.10 Lentiviral transfection

Lentiviral particles were purchased from GeneChem (Shanghai, China). Ishikawa and HEC-1A cells were seeded in 6-well plates and cultured for 24 h to reach ~30% confluence. Transduction was performed at manufacturer-recommended multiplicities of infection (MOI: Ishikawa=20, HEC-1A=10) with HitransG™ A infection enhancer (GeneChem, China). For JAML knockdown, stable cell lines were established through 7-day selection with puromycin (Beyotime, China) at concentrations of 2 μg/mL for Ishikawa and 2.5 μg/mL for HEC-1A cells. For JAML overexpression, cells were transduced with lentiviral particles encoding full-length human JAML cDNA and selected identically. To dissect functional interactions, JAML-overexpressing cells were subsequently transduced with lentiviral shRNA targeting STING (MOI matched to parental cell lines) and underwent dual antibiotic selection: puromycin (to maintain JAML overexpression) and blasticidin (5 μg/mL, InvivoGen, USA) for 7 days. Knockdown and overexpression efficiency was validated by Western blot analysis of JAML protein expression (33). The lentiviral shRNA sequences used were as follows: shJAML#1: 5′-CCCTGTTCTGATATTGATCGT-3′, shJAML#2: 5′-GAAGACTAATCCAGAGATAAA-3′. shSTING#1: 5′-GCATTACAACAACCTGCTACG-3′, shSTING#2: 5′- GCATCAAGGATCGGGTTTACA-3′.

### 2.11 Cell proliferation, migration, and invasion assays

Cell proliferation was assessed using CCK-8 kits (Dojindo, Japan) with 1.0×10^3^ cells/well seeded in 96-well plates. After 24 h pre-culture, 100 μL of serum-free medium containing 10% CCK-8 reagent was added at 0, 24, 48, and 72 h. Following 2 h incubation, absorbance at 450 nm was measured, with data normalized to 0 h values as relative cell viability (34).

Cell migration was evaluated by scratch wound healing and Transwell assays (35, 36). For scratch assays, confluent cells in 6-well plates (Corning, USA) were scratched to create 500 ± 50 μm wide wounds. After PBS washing, serum-free medium was added and wound closure was imaged at 0/24/48 h. Healing percentage was calculated as (A_0_-A_t_)/A_0_×100 using ImageJ (A_0_: initial width; A_t_: width at timepoint). For Transwell migration, cells were trypsinized, suspended, and seeded (4×10^4^ cells/insert) into Transwell chambers (8 μm pores; Corning, USA) with 20% FBS-DMEM as chemoattractant. After 24 h, migrated cells were methanol-fixed, stained with 1% crystal violet (Beyotime, China), and counted in ≥3 random 100× fields.

Invasion assays used Matrigel-coated Transwells. Inserts (8 μm pores) were pre-coated with 1:8 DMEM/Matrigel matrix and polymerized at 37 °C for 1 h. Cells (1×10^5^/chamber) were seeded in serum-free medium to upper chambers with 20% FBS-DMEM in lower chambers. After 24 h incubation, invaded cells were fixed with 4% paraformaldehyde, stained with 0.1% crystal violet, and quantified in ≥3 random 100× fields (36).

### 2.12 Preparation of conditioned media and analysis of macrophage polarization

Conditioned medium was prepared by collecting supernatants from Ishikawa and HEC-1A cells transduced with non-targeting shRNA (shNC) or JAML-targeting shRNAs (shJAML#1/shJAML#2). For Cyclic GMP-AMP (cGAMP) combination groups, cells were incubated with 3μM cGAMP (InvivoGen, USA, tlrl-nacga23) for 4 hours, followed by medium replacement and 6–8 hours of additional culture (37). Supernatants were centrifuged and filtered through 0.22μm membranes for subsequent experiments.

THP-1 cells were primed with 320 nM phorbol 12-myristate 13-acetate (PMA) for 6 h and then rested for 18 h to obtain M0 macrophages for subsequent polarization assays. Macrophages were cultured for 48 h at 37 °C under 5% CO_2_ in a 1:1 mixture of conditioned media and fresh RPMI-1640 complete medium to preserve secreted factors while replenishing depleted nutrients, thereby ensuring optimal viability and polarization (32). Cells were then stained with anti-human CD86-FITC (BioLegend, USA, 374204) and CD206-PE (BioLegend, USA, 321106) antibodies along with matched isotype controls for 30 min at 4°C in the dark. Flow cytometry was conducted using a CytoFLEX S instrument (Beckman Coulter, USA), with data analysis performed in cytExpert software.

### 2.13 Colony formation assay

For colony formation assays, Ishikawa and HEC-1A cells were seeded in 6-well plates at 3,000 cells/well. After adherence, the cGAMP combination group was pretreated with 3μM cGAMP (37). All groups, including cGAMP-pretreated and untreated controls, were then exposed to specified concentrations of paclitaxel (MedChemExpress, HY-B0015) or cisplatin (MedChemExpress, HY-17394) for 14 days (38, 39). Colonies were fixed with 4% paraformaldehyde for 20 min, stained with 0.1% crystal violet for 30 min, and those exceeding 50 μm in diameter were counted. All assays were performed in triplicate, with results expressed as mean ± standard deviation (40).

### 2.14 Subcutaneous xenograft model and treatment

After a one-week acclimation period, 4-week-old female BALB/c nude mice were randomly assigned to experimental groups (n=5 per group). EC xenograft models were established by subcutaneous injection of 1×10^6^ Ishikawa cells (stable transfectants of either shNC or shJAML suspended in 100 µL PBS) into the right thigh. For the cGAMP combination group, intratumoral injections of cGAMP (0.5 mg/kg) were initiated when tumor volumes reached approximately 50 mm^3^ and administered every 3 days. This dosing schedule was adapted from a published study reporting STING activation and antitumor activity with this regimen in a xenograft model (41). Control groups received equivalent volumes of PBS vehicle (42, 43).

Tumor progression monitoring began on day 4 post-inoculation, with body weight measured and tumor dimensions assessed using digital calipers every 3 days. Tumor volumes were calculated as V = 0.5 × length × width². At the experimental endpoint, mice were euthanized by cervical dislocation under sodium pentobarbital anesthesia prior to tumor resection. All experimental procedures were approved by the Animal Ethics Committee of Chongqing Medical University (IACUC-CQMU 2024-0991) and conducted in strict compliance with institutional guidelines for animal welfare and ethics.

### 2.15 Construction and validation of the nomogram model

A nomogram for predicting recurrence was developed using the institutional training cohort (n = 483) through multivariate Cox regression and externally validated in an independent cohort (n = 239). Predictor selection followed a two-step approach: univariate Cox regression first identified relapse-associated variables (JAML expression and clinicopathological parameters; P < 0.05), followed by forward stepwise selection in multivariate Cox regression to determine independent predictors (P < 0.05) (44).

The nomogram was constructed with R software (v4.3.2; survival and rms packages). Based on the training cohort’s ROC curve, the optimal risk threshold for 3-year RFS was determined by maximizing Youden’s index. Patients were stratified into high/low-risk groups, with between-group differences in RFS and OS compared via Kaplan-Meier curves (log-rank test). Calibration plots assessed agreement between predicted probabilities and actual recurrence rates (27).

### 2.16 Statistical analysis

Group differences were assessed using chi-square tests for categorical variables. For continuous variables, Student’s t-tests or Mann−Whitney U tests were used depending on whether data met assumptions of normality and equal variance (assessed by Shapiro−Wilk and Levene’s tests, respectively). All statistical analyses were performed using IBM SPSS Statistics (version 27.0) and GraphPad Prism (version 10.0). Experiments were independently repeated three times. Statistical significance was set at P < 0.05, and exact P values are reported where applicable.

## 3 Results

### 3.1 Downregulation of JAML in EC and its association with poor prognosis

**Figure 1:**
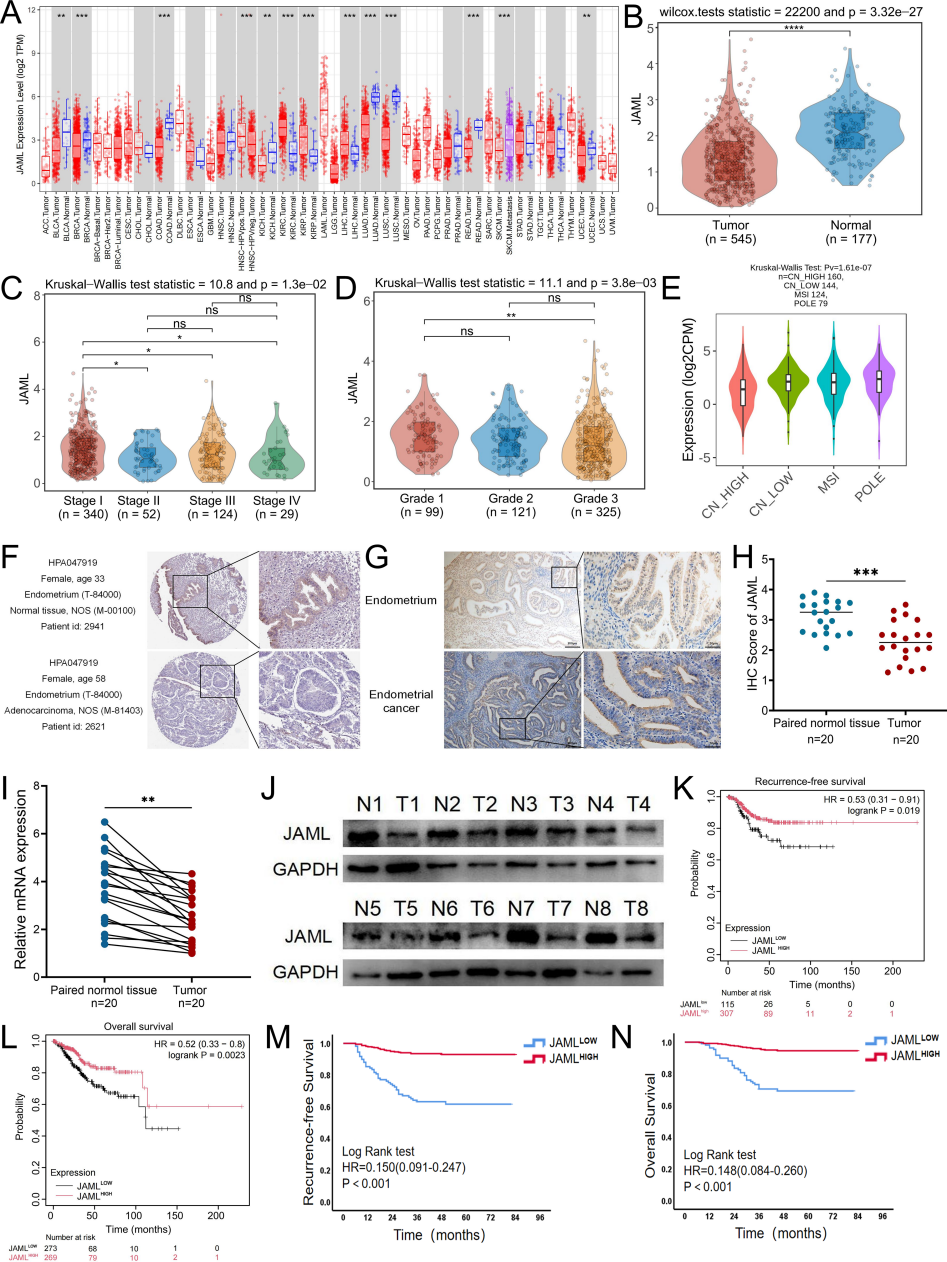
Expression and prognostic value of JAML in EC. **(A)** Pan-cancer analysis of JAML expression levels from the TCGA RNA-seq dataset. **(B)** Comparison of JAML expression between EC tissues and normal endometrial tissues. **(C, D)** JAML expression levels across EC samples stratified by FIGO stage and histological grade. **(E)** JAML expression profiles across the TCGA-defined molecular subtypes of EC. **(F, G)** Representative IHC images of JAML protein expression in normal endometrial and UCEC tissues. Images were obtained from the HPA database and our institutional cohort of 20 paired samples. **(H)** Quantitative IHC scores of JAML expression in paired adjacent non-tumor and tumor tissues from the institutional cohort. **(I)** JAML mRNA expression levels in 20 paired UCEC and adjacent normal tissues, as determined by RT-qPCR. **(J)** JAML protein expression levels in paired UCEC and adjacent normal tissues, as detected by western blot analysis. **(K, L)** Kaplan-Meier curves for RFS and OS in the TCGA cohort, stratified by JAML expression. **(M, N)** Kaplan-Meier curves for RFS and OS in the institutional cohort, stratified by JAML expression. Data are presented as mean ± SD. *p < 0.05, **p < 0.01, ***p < 0.001, ****p < 0.0001; ns, not significant.

Analysis of the TCGA pan-cancer dataset revealed significant downregulation of JAML in multiple solid tumors, including EC (**Figures 1A, B**). In EC, JAML expression decreased progressively with advanced FIGO stage and higher histologic grade (**Figures 1C, D**), with the lowest levels observed in the copy-number high (CN-H) molecular subtype (**Figure 1E**). IHC data from the HPA database and our institutional cohort showed JAML protein expression in tumor tissues compared to normal endometrium (**Figures 1F, G**). Consistent downregulation at both mRNA and protein levels was further validated in 20 matched tumor-normal pairs by IHC, qRT-PCR, and Western blot (**Figures 1H-J**).

In the TCGA-UCEC cohort, low JAML expression correlated significantly with advanced FIGO stage (P = 0.009), high tumor grade (P = 0.008), aggressive histologic subtypes (P = 0.001), TP53 mutation (P = 0.003), and administration of adjuvant radiotherapy (P = 0.044) (**Supplementary Table 4**). These associations were corroborated in our independent institutional cohort, where low JAML expression was additionally associated with advanced age (P = 0.013), deep myometrial invasion (P < 0.001), LVSI (P = 0.001), and receipt of adjuvant therapy (P = 0.001) (**Supplementary Table 5**).

Kaplan-Meier analysis further showed that in both cohorts, patients with high JAML expression had significantly longer RFS and OS compared to those with low expression. Specifically, in the TCGA cohort, the Hazard Ratio (HR) was 0.53 (P = 0.019) for RFS and 0.52 (P = 0.0023) for OS (**Figures 1K, L**); in the institutional cohort, the HR was 0.15 (P < 0.001) for RFS and 0.148 (P < 0.001) for OS (**Figures 1M, N**).

### 3.2 JAML regulates the malignant phenotypes of EC cells

**Figure 2:**
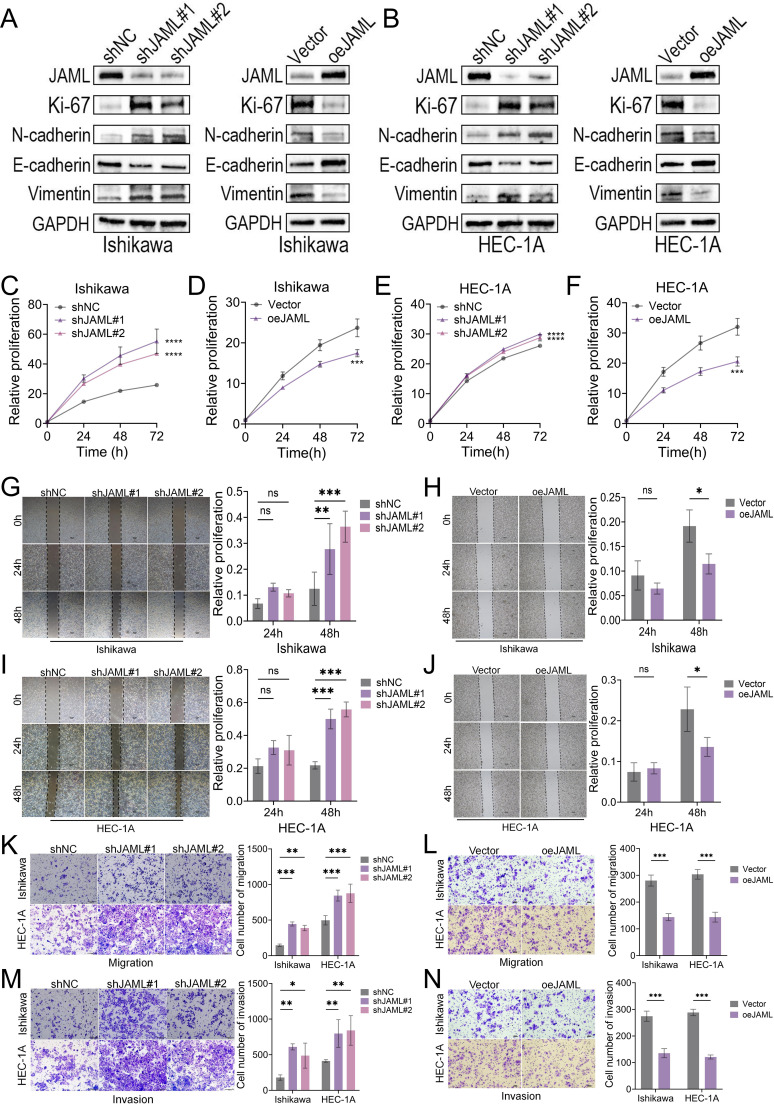
JAML regulates proliferation, migration, and invasion in EC cells. **(A, B)** Western blot analysis of JAML and EMT markers in Ishikawa and HEC-1A cells following JAML knockdown or overexpression. **(C, D)** CCK-8 assay assessing proliferation of Ishikawa cells following JAML knockdown or overexpression. **(E, F)** CCK-8 assay assessing proliferation of HEC-1A cells following JAML knockdown or overexpression. **(G, H)** Wound healing assay evaluating migration of Ishikawa cells after JAML knockdown or overexpression. **(I, J)** Wound healing assay evaluating migration of HEC-1A cells after JAML knockdown or overexpression. **(K, L)** Transwell migration assay for Ishikawa and HEC-1A cells following JAML knockdown or overexpression. **(M, N)** Transwell invasion assay for Ishikawa and HEC-1A cells following JAML knockdown or overexpression. Data are presented as mean ± SD. *p < 0.05, **p < 0.01, ***p < 0.001, ****p < 0.0001; ns, not significant.

Stable knockdown and overexpression models of JAML were established in Ishikawa and HEC-1A cell lines via lentiviral transduction (**Supplementary Figure 1**). Western blot analysis showed that JAML knockdown upregulated the proliferation marker Ki-67 and promoted epithelial−mesenchymal transition (EMT), characterized by increased N-cadherin and vimentin and decreased E-cadherin. Conversely, JAML overexpression suppressed these EMT markers (**Figures 2A, B**). Functionally, JAML knockdown enhanced malignant phenotypes, whereas overexpression inhibited them. CCK−8 assays showed that knockdown promoted proliferation in both lines, while overexpression suppressed it (**Figures 2C–F**). In scratch assays, knockdown accelerated wound closure, and overexpression delayed it (**Figures 2G–J**). Transwell migration assays confirmed that knockdown increased migrated cell numbers, while overexpression decreased them (**Figures 2K–N**). These results demonstrate that JAML negatively regulates proliferation, migration, and EMT in EC cells.

### 3.3 JAML expression correlates with immune infiltration and macrophage polarization in EC

**Figure 3:**
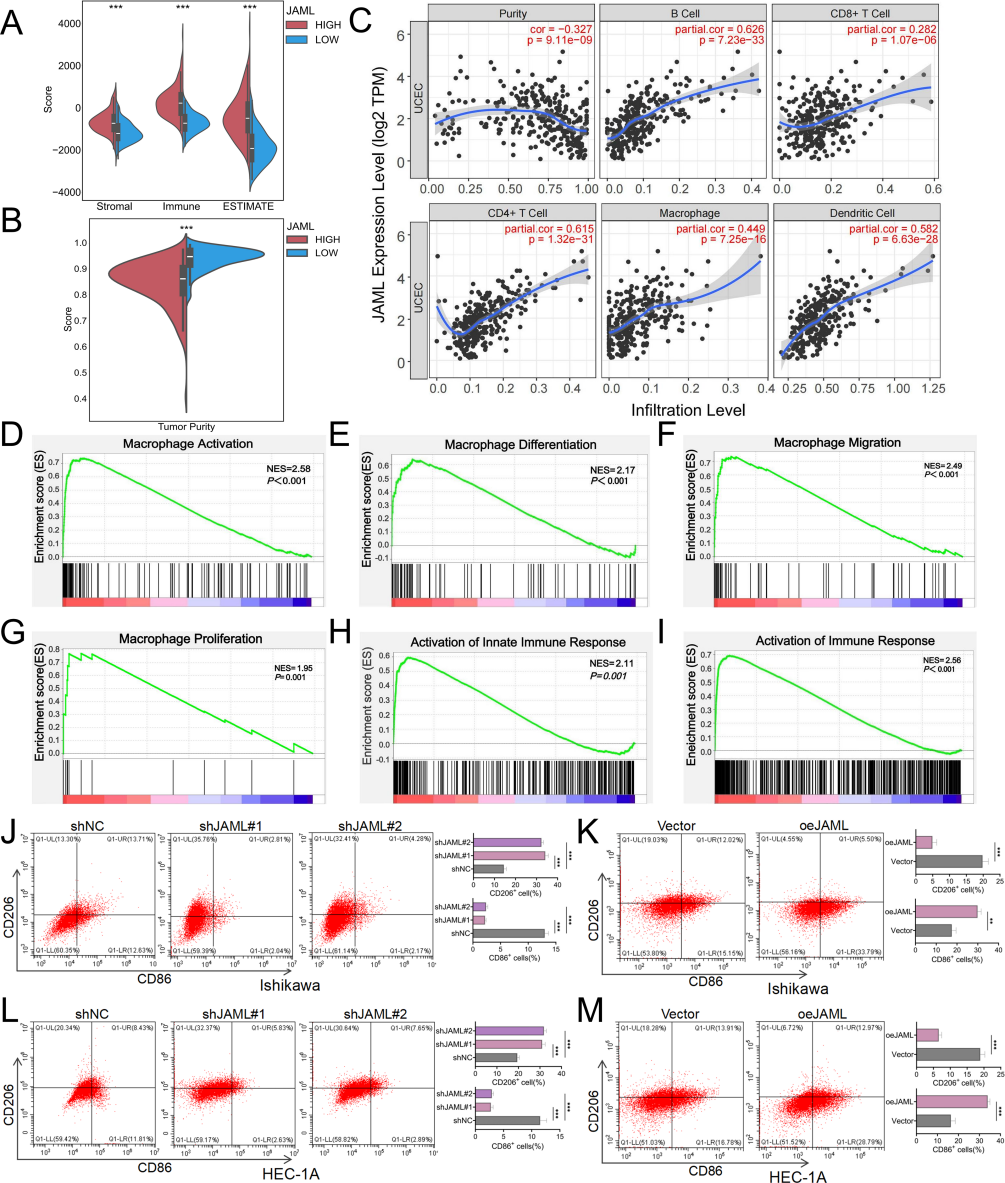
JAML modulates the tumor immune microenvironment and macrophage polarization in EC. **(A, B)** Association between JAML expression and stromal, immune, and ESTIMATE scores as well as tumor purity in UCEC samples. **(C)** Correlations between JAML expression and tumor purity or immune cell infiltration levels in UCEC (TIMER database). **(D–I)** GSEA performed on the TCGA-UCEC dataset demonstrates significant enrichment of gene sets related to: macrophage activation, macrophage differentiation, macrophage migration, macrophage proliferation, innate immune response activation, and immune response activation. **(J, K)** Flow cytometric analysis of CD86^+^ M1-like and CD206^+^ M2-like macrophages treated with conditioned media from Ishikawa cells with JAML knockdown or overexpression. **(L, M)** Flow cytometric analysis of CD86^+^ M1-like and CD206^+^ M2-like macrophages treated with conditioned media from HEC-1A cells with JAML knockdown or overexpression. Data are presented as mean ± SD. *p < 0.05, **p < 0.01, ***p < 0.001, ****p < 0.0001; ns, not significant.

ESTIMATE algorithm analysis revealed that EC samples with high JAML expression exhibited significantly elevated stromal, immune, and composite scores (all P < 0.001), along with reduced tumor purity (P < 0.001), indicating an immune/stroma-rich microenvironment (**Figures 3A, B**). TIMER analysis demonstrated positive correlations between JAML expression and infiltration levels of B cells (r = 0.626, P = 7.23e-33), CD4^+^ T cells (r = 0.615, P = 1.32e-31), and macrophages (r = 0.449, P = 7.25e-16) (**Figure 3C**), further validated by MCP-counter quantification (**Supplementary Figure 2A**).

Single-cell RNA sequencing showed JAML enrichment predominantly in myeloid cells (monocytes/macrophages, dendritic cells), while it was undetectable in lymphoid subsets or fibroblasts (**Supplementary Figures 2B, C**). GSEA revealed significant activation of immune-related pathways in the high-JAML group, including macrophage activation, differentiation, migration, proliferation, and innate immune response (all P < 0.001; **Figures 3D–I**). Extended TCGA analysis confirmed positive correlations between JAML expression and tumor-infiltrating lymphocyte markers, MHC class II, and co-stimulatory molecules (**Supplementary Figure 3**). Functional validation demonstrated that conditioned medium from JAML−knockdown EC cells induced a shift in macrophage polarization, characterized by a decrease in CD86^+^ M1−like cells and an increase in CD206^+^ M2−like cells (P < 0.001; **Figures 3J, L**). Conversely, conditioned medium from JAML−overexpressing EC cells promoted an opposite polarization shift, marked by an increase in CD86^+^ M1−like cells and a decrease in CD206^+^ M2−like cells (P < 0.01; **Figures 3K, M**).

### 3.4 JAML modulates macrophage polarization via cGAS-STING signaling in EC

**Figure 4:**
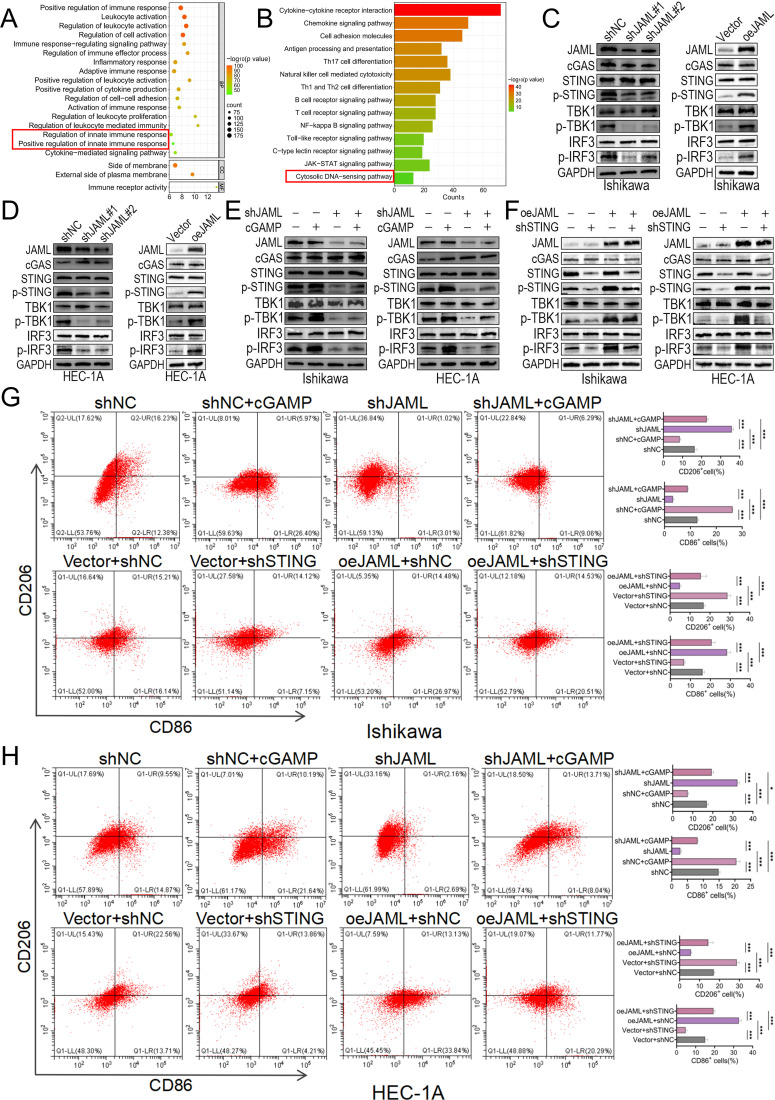
Functional characterization of JAML in cGAS-STING signaling in EC cells. **(A, B)** GO term and KEGG pathway enrichment analyses of JAML-coexpressed genes. **(C)** Western blot analysis of JAML, cGAS, STING, TBK1, and IRF3 expression, and phosphorylation of STING, TBK1, and IRF3 in Ishikawa cells following JAML knockdown or overexpression. **(D)** Western blot analysis of JAML, cGAS, STING, TBK1, and IRF3 expression, and phosphorylation of STING, TBK1, and IRF3 in HEC−1A cells following JAML knockdown or overexpression. **(E)** Western blot analysis of cGAS, STING, TBK1, and IRF3 expression, and phosphorylation of STING, TBK1, and IRF3, in JAML-knockdown Ishikawa and HEC-1A cells treated with or without the STING agonist cGAMP. **(F)** Western blot analysis of cGAS, STING, TBK1, and IRF3 expression, and phosphorylation of STING, TBK1, and IRF3, in JAML-overexpressing Ishikawa and HEC-1A cells with or without concomitant STING knockdown. **(G, H)** Flow cytometric analysis of macrophage polarization. M0 macrophages were treated with conditioned media from Ishikawa or HEC-1A cells under indicated manipulations. Loss-of-function: Cells transfected with shNC or shJAML, with or without cGAMP. Gain-of-function: Cells transfected with Vector or oeJAML, combined with shNC or shSTING. Proportions of CD86^+^ M1-like and CD206^+^ M2-like subsets are shown. Note: Data are presented as mean ± SD. *p < 0.05, **p < 0.01, ***p < 0.001, ****p < 0.0001; ns, not significant.

Based on the GeneMANIA protein interaction network analysis, JAML is predicted to have functional associations with immune-related molecules, including CXADR, ITGAX, and ITGB2 (**Supplementary Figure 4A**). Consistently, in the TCGA−UCEC cohort, JAML expression was significantly positively correlated with key innate immune genes such as IL2RG and PTPN7 (**Supplementary Figure 4B**). GO/KEGG enrichment analyses revealed that genes co-expressed with JAML in EC are significantly enriched in immune and inflammatory processes, including innate immune regulation, the cytosolic DNA-sensing pathway, and NF-κB signaling (**Figures 4A, B**). Since the cGAS-STING pathway serves as both a central component of innate immunity and the primary sensor for cytosolic DNA, we focused our investigation on this pathway to examine its functional relationship with JAML (45). Experimental results demonstrated that JAML bidirectionally modulated phosphorylation of key cGAS−STING components (STING, TBK1, IRF3) without affecting their total protein levels (**Figures 4C, D**). Specifically, JAML knockdown−induced phospho−suppression was partially rescued by cGAMP treatment, whereas JAML overexpression−enhanced phosphorylation was partially reversed by concomitant STING knockdown (with knockdown efficiency validated in **Supplementary Figure 5**) (**Figures 4E, F**). Conditioned medium from JAML-knockdown EC cells shifted macrophage polarization from the CD86^+^ M1 toward the CD206^+^ M2 phenotype; this shift was attenuated when the knockdown cells were treated with cGAMP. Conversely, medium from JAML-overexpressing cells promoted a shift from M2 to M1, and this polarization shift was partially blocked by concomitant STING knockdown (**Figures 4G, H**).

**Figure 5:**
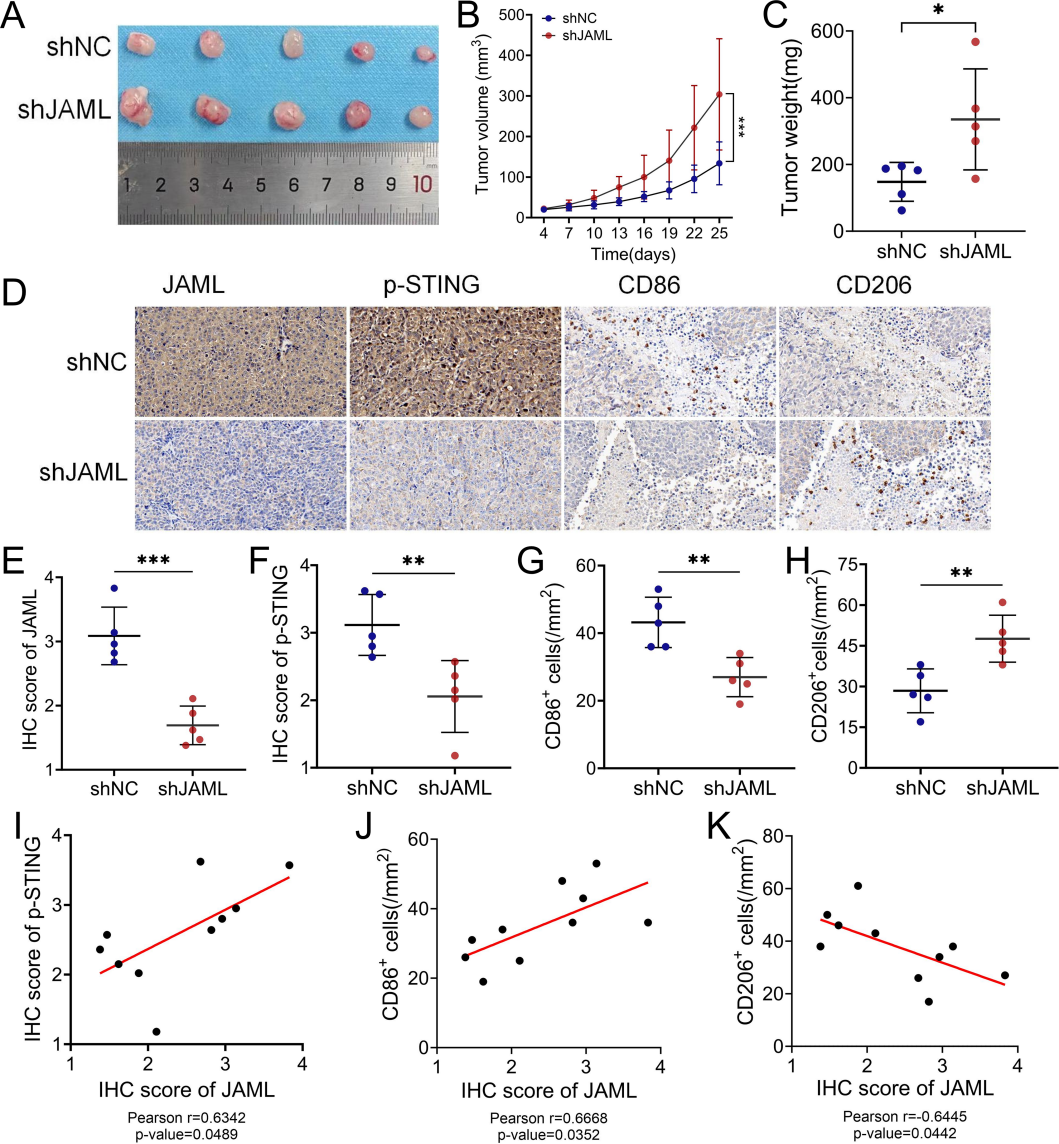
JAML knockdown promotes tumor growth and macrophage polarization in EC *in vivo*. **(A)** Xenograft tumors from shNC and shJAML groups. **(B)** Tumor growth curves. **(C)** Final tumor weights. **(D)** Representative IHC staining of JAML, p-STING, CD86, and CD206. **(E, F)** Quantitative IHC analysis of JAML and p-STING expression in shNC versus shJAML tumors. **(G, H)** Quantification of CD86^+^ M1-like and CD206^+^ M2-like macrophage infiltration in shNC versus shJAML tumors. **(I-K)** Correlation analyses between JAML expression levels and p-STING IHC scores, CD86^+^ cell density, and CD206^+^ cell density across all tumor samples. Note: Data are presented as mean ± SD. *p < 0.05, **p < 0.01, ***p < 0.001, ****p < 0.0001; ns, not significant.

**Figure 6:**
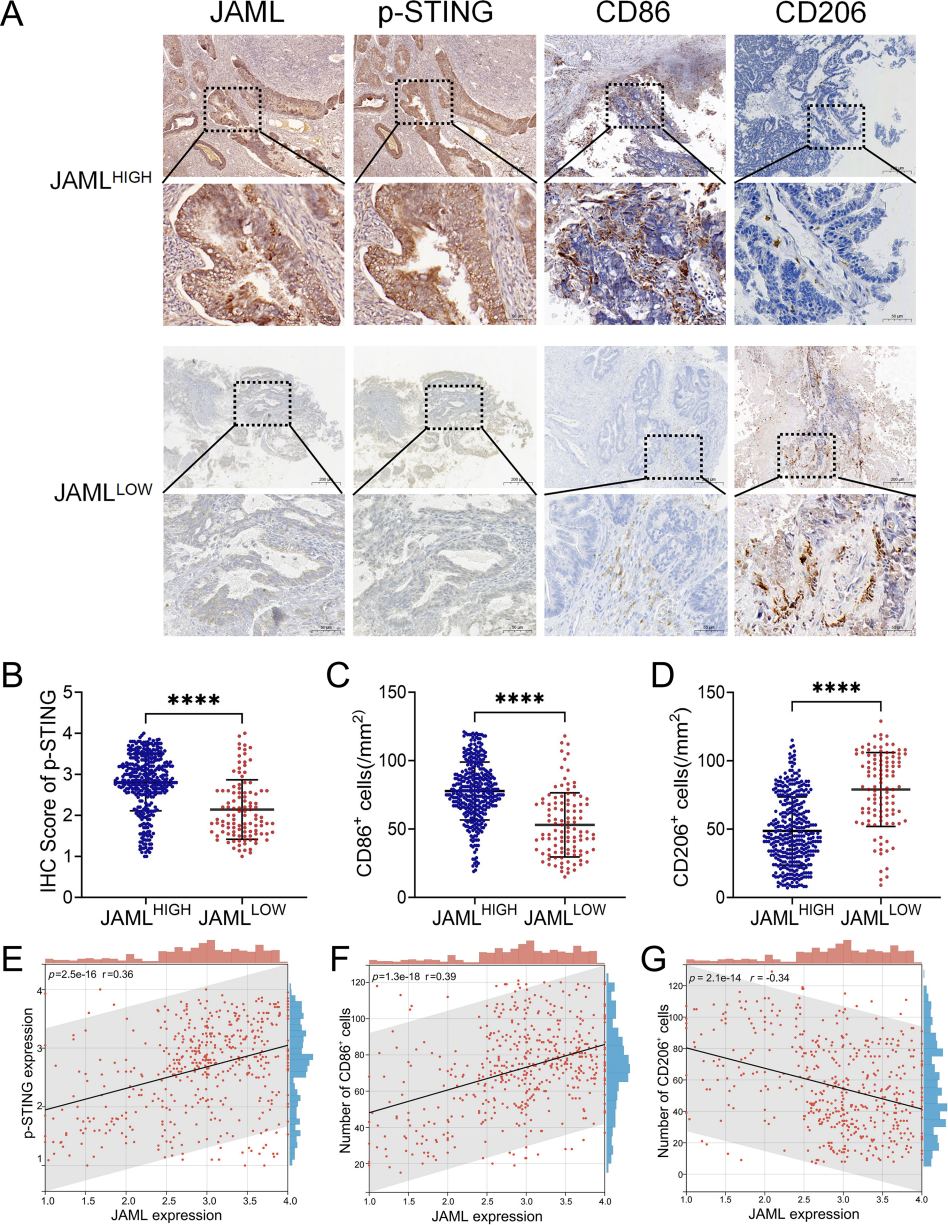
Correlation of JAML expression with p-STING levels and macrophage polarization. **(A)** Representative IHC staining of p-STING, CD86, and CD206 in human EC tissue samples exhibiting high versus low JAML expression levels. **(B-D)** Comparison of p-STING levels, CD86^+^ cells, and CD206^+^ cells between high and low JAML expression groups. **(E-G)** Correlation of JAML expression with p-STING levels, CD86^+^ cells, and CD206^+^ cells. Note: Data are presented as mean ± SD. *p<0.05, **p<0.01, ***p<0.001, ****p<0.0001; n.s., not significant.

Consistent with its tumor-promoting role, JAML knockdown in subcutaneous Ishikawa xenografts significantly accelerated tumor growth (P < 0.001) and increased terminal tumor burden (P < 0.05) (**Figures 5A–C**). This was accompanied by a tumor microenvironment characterized by reduced p-STING and CD86^+^ cells alongside elevated CD206^+^ cells (P < 0.01; **Figures 5D-H**). Correlation analyses revealed that intratumoral JAML expression positively correlated with p-STING (r = 0.6342, P = 0.0489) and CD86^+^ cell density (r = 0.6668, P = 0.0352) but negatively correlated with CD206^+^ density (r = –0.6445, P = 0.0442, **Figures 5I–K**). Importantly, analysis of 483 clinical EC samples showed that low JAML expression was associated with significantly reduced p−STING levels and CD86^+^ cell density, alongside increased CD206^+^ cell density (all P < 0.0001; **Figures 6A–D**). Correlation analysis further indicated moderate positive correlations between JAML expression and both p−STING levels (r = 0.36) and CD86^+^ cell density (r = 0.39), and a moderate inverse correlation with CD206^+^ cell density (r = –0.34; all P < 0.0001; **Figures 6E–G**).

### 3.5 JAML expression as a potential biomarker for treatment sensitivity in EC

**Figure 7:**
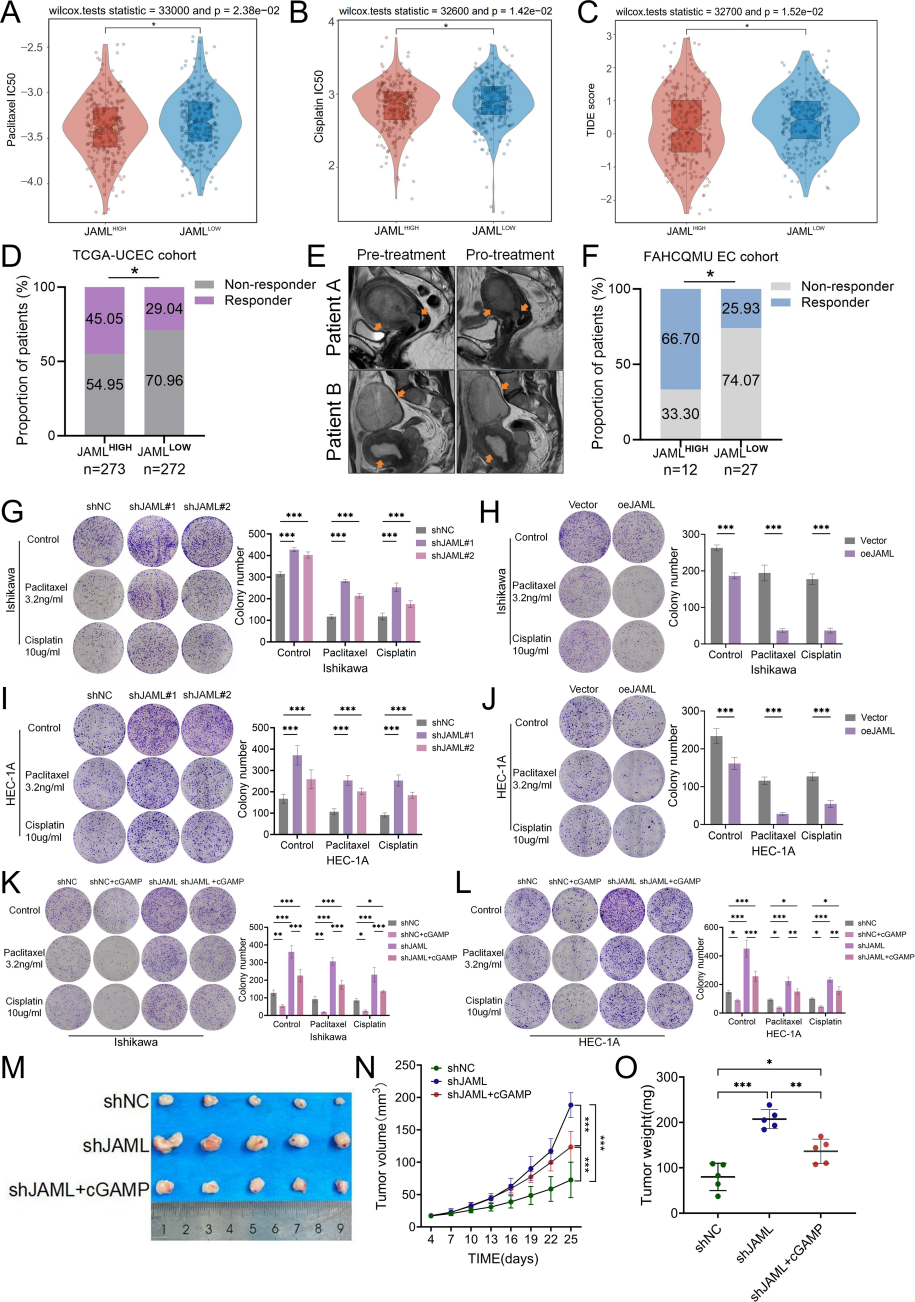
JAML modulates therapy response and tumor growth via the STING pathway in EC. **(A, B)** Differential sensitivity to paclitaxel and cisplatin in high- versus low-JAML expression groups from the GDSC database. **(C, D)** Analysis of predicted immunotherapy response using the TIDE algorithm, showing TIDE scores and responder distribution between high- and low-JAML expression groups. **(E)** Representative MRI images from two JAML-high patients in the FAHCQMU cohort, showing tumor regions (orange arrows) before and after camrelizumab-based therapy. **(F)** Association between tumor JAML expression and objective response to the camrelizumab-based regimen in the FAHCQMU EC cohort. **(G, H)** Colony-formation assays in Ishikawa cells after JAML knockdown or overexpression, followed by chemotherapy treatment. **(I, J)** Colony-formation assays in HEC-1A cells after JAML knockdown or overexpression, followed by chemotherapy treatment. **(K, L)** Effect of STING agonist cGAMP on colony formation in Ishikawa and HEC-1A cells transduced with shNC or shJAML. **(M–O)***In vivo* validation of therapy: Tumor images from each group, tumor growth curves, and final tumor weights for shNC, shJAML, and shJAML + cGAMP groups. Note: Data are presented as mean ± SD. *p < 0.05, **p < 0.01, ***p < 0.001, ****p < 0.0001; ns, not significant.

EC is primarily adenocarcinoma, with combined immunotherapy and chemotherapy serving as the standard treatment for advanced and recurrent cases (46). Utilizing data from the GDSC database, this study evaluated the correlation between JAML expression levels and sensitivity to conventional chemotherapeutic agents. Results indicated that high JAML expression was associated with increased sensitivity to cisplatin and paclitaxel (both p < 0.05; **Figures 7A, B**). In addition, predictive analysis using the TIDE algorithm suggested a higher likelihood of immunotherapy response in patients with high JAML expression (45.05% vs. 29.04%, p = 0.0152; **Figures 7C, D**). This bioinformatic finding received preliminary support from an independent clinical cohort (n=39) at our center, which enrolled patients treated with neoadjuvant chemotherapy combined with the PD-1 inhibitor camrelizumab. Representative Magnetic Resonance Imaging (MRI) images illustrated partial tumor shrinkage in JAML−high patients following camrelizumab−based therapy (**Figure 7E**). In this cohort, high JAML expression was also associated with a significantly higher objective response rate (ORR: 66.7% vs. 25.9%, P = 0.020; **Figure 7F**) and a longer median progression−free survival (PFS: 31.0 vs. 11.0 months, P = 0.027; **Supplementary Table 6**). Given balanced baseline characteristics, these efficacy differences may be attributable to JAML expression levels.

Colony formation assays showed that shJAML cells formed more colonies than shNC controls under paclitaxel (3.2 ng/ml) or cisplatin (10 μg/ml) treatment (**Figures 7G, I**), aligning with GDSC data and suggesting that JAML loss promotes chemoresistance. Conversely, JAML overexpression reduced colony formation under the same conditions (**Figures 7H, J**), consistent with enhanced chemosensitivity. To elucidate whether JAML regulates chemosensitivity through the STING pathway, we further evaluated the rescuing effect of cGAMP on chemoresistance in JAML-knockdown cells (**Figures 7K, L**). cGAMP treatment groups were established for both shNC and shJAML cells. Untreated shJAML cells showed the highest colony counts, while untreated shNC cells showed the lowest. cGAMP treatment further increased the sensitivity of shNC cells, reducing their colony count to an even lower level. Similarly, cGAMP partially reversed chemoresistance in shJAML cells, significantly decreasing their colony count compared to untreated shJAML cells. Importantly, however, even with cGAMP treatment, the colony count of shJAML cells remained significantly higher than that of shNC cells (with or without cGAMP), indicating that cGAMP cannot fully overcome JAML deficiency-induced chemoresistance or restore sensitivity to the level of shNC cells. These results collectively suggest that JAML regulates chemosensitivity not only through the STING pathway but also via additional, STING-independent mechanisms.

*In vivo* assays corroborated the tumor-promoting effect of JAML knockdown: shJAML-bearing mice showed significantly accelerated tumor growth and increased terminal tumor burden relative to controls (both volume and weight, p < 0.001), whereas cGAMP treatment (0.5 mg/kg every 3 days) partially reversed this enhancement (**Figures 7M–O**). No significant body weight loss was observed across all groups throughout the treatment period, indicating good tolerability of the regimen (**Supplementary Figure 6**).

### 3.6 Construction of a recurrence prediction model for EC based on JAML and clinicopathological parameters

To develop and validate a prognostic model integrating JAML expression, the cohort from FAHCQMU was utilized as the training set (n=483), while 239 EC patients retrospectively enrolled from WCHCQMU served as the validation cohort. The two groups showed comparable baseline characteristics across all recorded clinicopathological parameters (P > 0.05; **Supplementary Table 7**). On multivariate Cox regression analysis of the training cohort, which included variables significant in univariate analysis, JAML was identified as an independent predictor of EC recurrence after adjustment for clinicopathological confounders. Specifically, high JAML expression was significantly associated with improved RFS, conferring a substantial risk reduction (HR = 0.225, 95% CI: 0.132-0.383, P < 0.001; **Supplementary Figure 7**). Other established factors, including age, FIGO stage, myometrial invasion depth, histological subtype, and P53 status, also remained significant (all P < 0.05).

**Figure 8:**
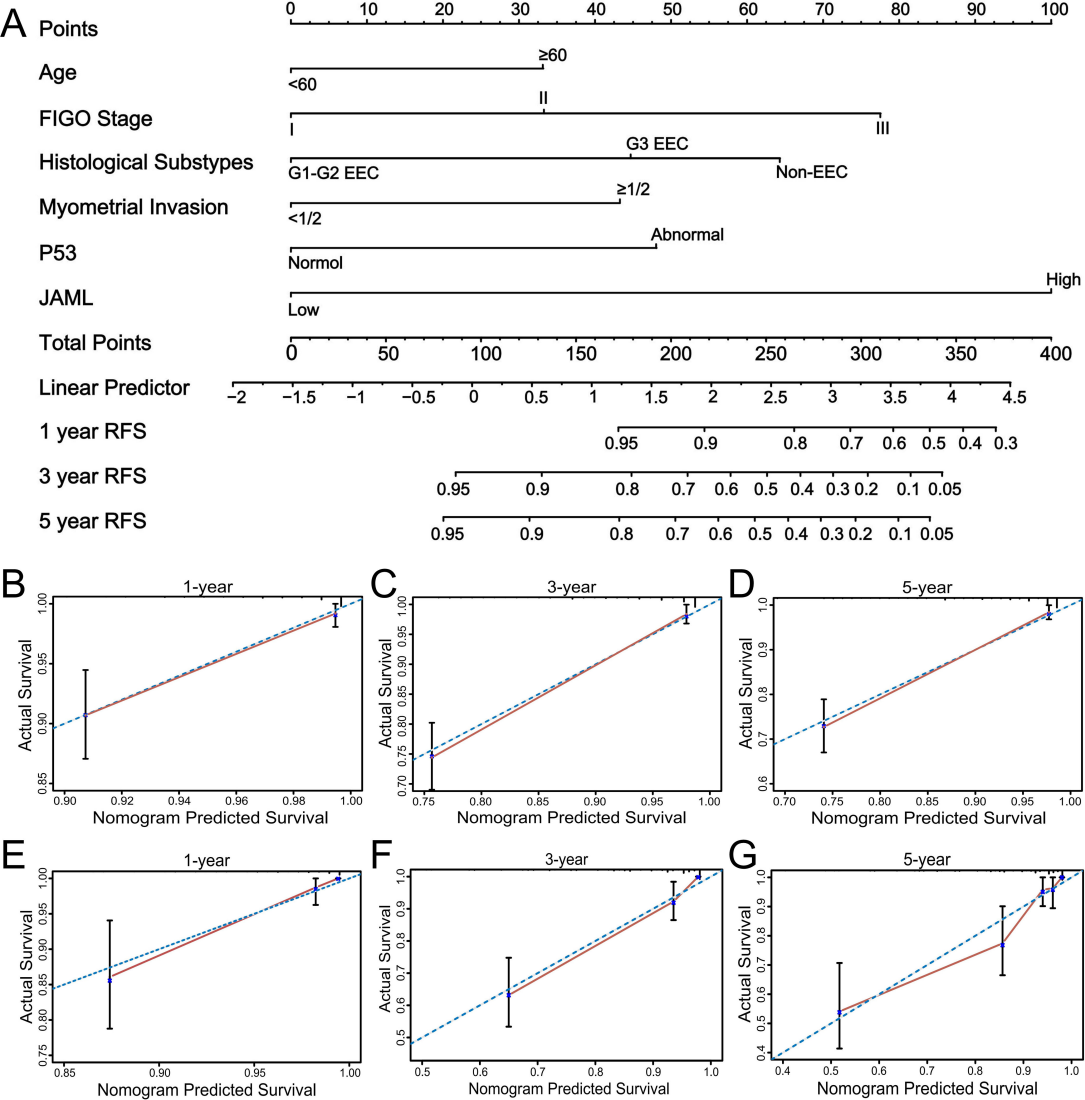
Development and validation of a JAML-based nomogram for predicting RFS in EC patients. **(A)** A prognostic nomogram for predicting 1-, 3-, and 5-year RFS. The model incorporates clinical parameters (Age, FIGO Stage, Histological Subtype, Myometrial Invasion, P53 status) and JAML expression level. Usage: For each patient, locate the corresponding point on the ‘Points’ axis for every variable. Sum all assigned points to get the ‘Total Points’. Finally, project the total points down to the survival probability axes at the bottom to obtain the predicted 1-, 3-, and 5-year RFS rates. **(B-D)** Calibration curves for the nomogram from internal validation, assessing the agreement between predicted and observed 1-year, 3-year, and 5-year RFS. The dashed diagonal line represents an ideal prediction. **(E-G)** Calibration curves for the nomogram from external validation, assessing the agreement between predicted and observed 1-year, 3-year, and 5-year RFS. The dashed diagonal line represents an ideal prediction.

The integrated prognostic model combining JAML expression with clinicopathological parameters demonstrated superior discriminative capability compared to single-component models. In the training cohort, the combined model achieved an AUC of 0.885 versus 0.730 for JAML alone and 0.830 for clinicopathological factors alone. This advantage persisted in the validation cohort (AUC: 0.852 vs 0.657 vs 0.793; **Supplementary Figure 8**). In the final nomogram comprising six variables, JAML demonstrated considerable predictive weight (**Figure 8A**).

The nomogram demonstrated excellent calibration accuracy across 1-, 3-, and 5-year timepoints in both internal and external validation (**Figures 8B–G**). Using the optimal risk−score threshold of 0.882 determined by maximizing Youden’s index in ROC analysis for predicting 3−year recurrence (**Supplementary Figure 9**), patients were stratified into low−risk (nomogram score > 0.882) and high−risk (nomogram score ≤ 0.882) groups. Kaplan-Meier analysis confirmed significantly prolonged recurrence-free survival in the low-risk group across both cohorts (**Supplementary Figure 10**).

## 4 Discussion

The global incidence of EC continues to rise (47). Meanwhile, patients with advanced, recurrent, or metastatic disease face limited treatment options and generally poor clinical outcomes (48). Recently, immunotherapy combined with chemotherapy has emerged as a new therapeutic option for this population. However, owing to the heterogeneity of the tumor microenvironment, only a subset of patients derives lasting benefit (49). Consequently, identifying robust biomarkers that can predict prognosis and guide treatment selection remains a major challenge in optimizing the management of advanced EC.

This study provides initial evidence that JAML expression may serve as a complementary diagnostic and prognostic biomarker in EC. Functionally, knockdown of JAML enhanced tumor cell proliferation, migration, and invasion, suggesting a tumor-suppressive role in this context. Given the recognized importance of the tumor microenvironment (TME) in shaping tumor progression and therapeutic outcomes (50), we investigated the relationship between JAML expression and the immune context of EC. Our preliminary analysis revealed that JAML expression correlates positively with macrophage infiltration levels, as well as with stromal and immune scores—metrics reflecting non-tumor compartment activity and potential prognostic value in cancer (51). Notably, the observed tumor-suppressive function of JAML in EC contrasts with its reported oncogenic roles in gastrointestinal malignancies such as gastric and colorectal cancers (14). This functional discrepancy may be attributable to tissue-specific factors or differences in the TME.

To clarify how JAML regulates the tumor immune microenvironment, we analyzed public transcriptomic data, which revealed its close association with macrophage−related functional pathways. This finding was further validated experimentally, showing that JAML expression bidirectionally regulates macrophage polarization: knockdown of JAML promoted a shift toward an M2−like phenotype, whereas overexpression induced an M1−like phenotype. This regulatory process appears to involve the cGAS–STING pathway, as the STING agonist cGAMP partially reversed the M2 polarization induced by JAML knockdown, and STING knockdown partially reduced the M1 polarization induced by JAML overexpression. In a clinical cohort of 438 samples, JAML exhibited statistically significant but moderate correlations with p−STING and macrophage markers (CD86 and CD206). This likely reflects considerable tumor heterogeneity, where STING activation and macrophage polarization are co−regulated by multiple factors beyond JAML.

Although immune checkpoint inhibitors (ICIs) have significantly improved the treatment landscape for advanced cancers, objective response rates remain between 15% and 60%, leaving a substantial proportion of patients without meaningful clinical benefit (52). Identifying predictive biomarkers is therefore crucial to avoid ineffective treatments, minimize associated toxicities, and enhance cost-effectiveness (53, 54). Established biomarkers include PD-L1 expression, TME immune composition, tumor neoantigen burden, genetic/epigenetic features, and the microbiome (55). Analysis using the TIDE algorithm indicated that patients with high JAML expression had a significantly higher estimated objective response rate to immunotherapy. This analytical finding received preliminary support from our clinical cohort (n=39), which included patients receiving neoadjuvant therapy combined with the PD-1 inhibitor camrelizumab. In this cohort, high JAML expression correlated with significantly higher ORR and longer PFS. Although regimen heterogeneity precludes definitive conclusions for ICI monotherapy, these initial data suggest JAML may be a biomarker worthy of further investigation in EC. In conventional chemotherapy, the combination of paclitaxel and platinum serves as the backbone regimen for EC patients, but its efficacy is markedly influenced by intertumoral heterogeneity (56). Analysis of the GDSC database revealed that high JAML expression is significantly associated with increased sensitivity to paclitaxel and cisplatin in EC, as evidenced by a marked reduction in IC50 (P < 0.01). Functional analysis further revealed that JAML knockdown significantly diminished the inhibitory effects of both chemotherapeutic agents on EC cells, while overexpression conversely enhanced their efficacy. Notably, the STING agonist cGAMP partially reversed the drug-tolerant phenotype induced by JAML down-regulation. The incomplete reversal points to cGAS–STING involvement in chemosensitivity, while also suggesting possible STING-independent mechanisms. Collectively, JAML expression may act as an upstream regulator influencing EC sensitivity to both immunotherapy and chemotherapy. Therefore, JAML-low tumors may represent a candidate population for future clinical exploration.

This study reveals the tumor-intrinsic tumor-suppressive and immunomodulatory functions of JAML in EC, though several limitations should be noted. First, while the immunodeficient models used helped analyze tumor cell–macrophage interactions, they could not assess the role of JAML in adaptive immunity, such as CD8^+^ T-cell responses. Moreover, whether the cGAMP-mediated rescue persists in immunocompetent settings requires further validation. Second, experiments indicate that alterations in JAML expression within tumor cells influence macrophage polarization. However, the mechanisms by which JAML regulates STING phosphorylation, as well as the key downstream mediators through which STING governs macrophage polarization, remain unclear and warrant further experimental investigation. Third, although single-cell sequencing suggests myeloid cells as a major source of JAML in the tumor microenvironment, their cell-autonomous functions and specific contributions to the immune landscape warrant further investigation using cell type-specific knockdown models.

## 5 Conclusion

This study confirms that JAML expression levels may serve as an auxiliary diagnostic and prognostic biomarker for EC, with its low expression being associated with unfavorable clinical outcomes. Mechanistically, JAML exerts its tumor−suppressive function by modulating the tumor microenvironment and inhibiting phenotypes that promote tumor progression. Furthermore, JAML expression can help predict patient responses to immune checkpoint inhibitors and conventional chemotherapy, providing a reference for clinical individualized treatment.

## Data Availability

The original contributions presented in the study are included in the article/**Supplementary Materials**. Further inquiries can be directed to the corresponding author.

## Supplementary material

Supplementary Figure 1 JAML knockdown and overexpression efficiency validation.

Supplementary Figure 2 JAML expression correlates with tumor microenvironment features in EC.

Supplementary Figure 3 Pan-cancer correlation analysis of JAML with immune features.

Supplementary Figure 4 JAML co-expression networks.

Supplementary Figure 5 STING knockdown efficiency validation.

Supplementary Figure 6 Body weight monitoring during treatment.

Supplementary Figure 7 Forest plots of Cox regression analyses for RFS in the training cohort.

Supplementary Figure 8 Predictive performance of JAML, clinicopathological parameters, and their combination for RFS.

Supplementary Figure 9 ROC curve of the nomogram for predicting 3-year RFS in EC.

Supplementary Figure 10 Kaplan-Meier survival analysis based on the nomogram-defined risk groups.

Supplementary Table 1 Bioinformatics tools and databases used in this study.

Supplementary Table 2 Inter-observer agreement for JAML expression by Cohen’s kappa in training and validation cohorts.

Supplementary Table 3 Inter-observer reliability of continuous immunohistochemistry assessments by ICC.

Supplementary Table 4 Association between JAML expression and clinicopathological characteristics in the UCEC cohort.

Supplementary Table 5 Association between JAML expression and clinicopathological characteristics of EC in the training cohort (N = 483).

Supplementary Table 6 Comparison of Baseline Characteristics and Treatment Outcomes in Patients with Advanced or Recurrent EC Stratified by JAML Expression Level (N = 39).

Supplementary Table 7 Clinicopathological Characteristics of Patients with EC in the Training and Validation Cohorts.
