# Supplementary material for: The prognostic significance of JAML and its role in remodeling the immune microenvironment via the cGAS-STING pathway in endometrial cancer
Source: Front Immunol. 2026 Jan 29;17:1738596. doi: 10.3389/fimmu.2026.1738596 (PMC12894416; doi:10.3389/fimmu.2026.1738596)
Supplement: Supplementary Figure 1 — JAML knockdown and overexpression efficiency validation. [file Image1.pdf]

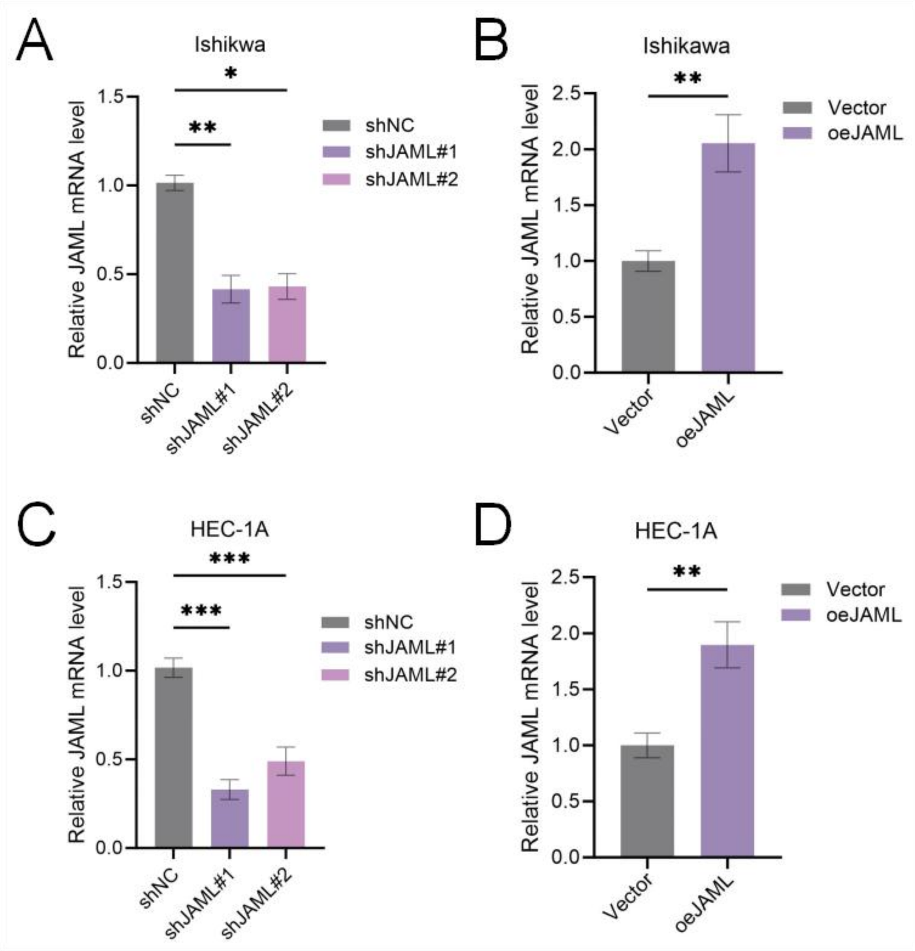

Supplementary Figure 1

JAML knockdown and overexpression efficiency validation. JAML mRNA expression in Ishikawa cells transfected with shJAML or oeJAML. (C, D) JAML mRNA expression in HEC-1A cells transfected with shJAML or oeJAML. Note: Data are presented as mean ± SD. *$p < 0.05$, **$p < 0.01$, ***$p < 0.001$; ns, not significant.

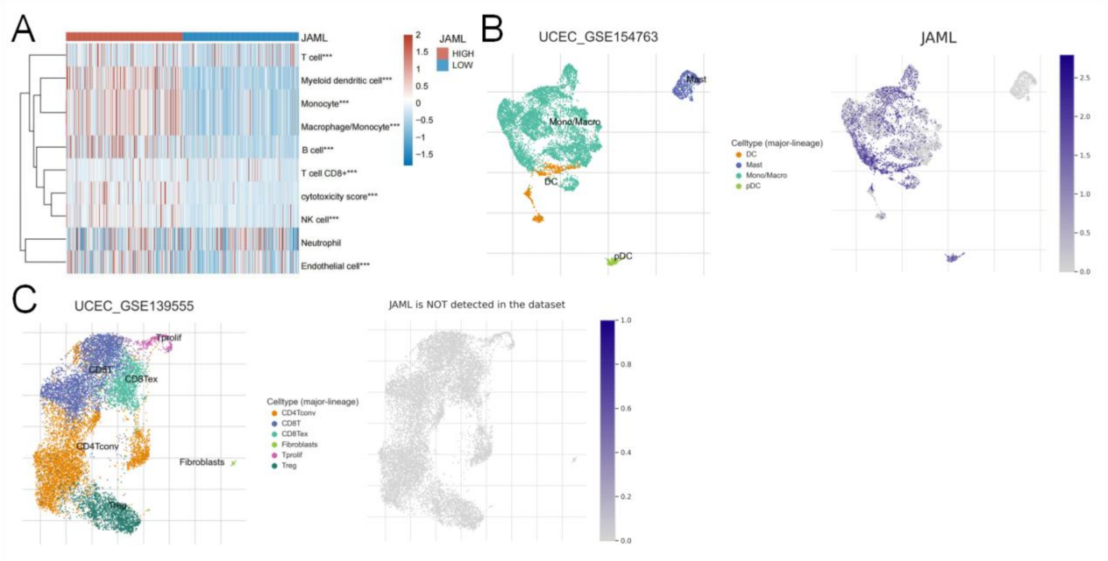

Supplementary Figure 2

JAML expression correlates with tumor microenvironment features in EC. (A) Correlation analysis between JAML expression and the abundance of various immune and stromal cells in the TCGA-UCEC cohort. (B, C) Comparison of the JAML gene signature expression between a UCEC datasets GSE154763 (B) and GSE139555 (C) from the TISCH database, demonstrating its heterogeneous expression across studies. $*p < 0.05$, $**p < 0.01$, $***p < 0.001$.

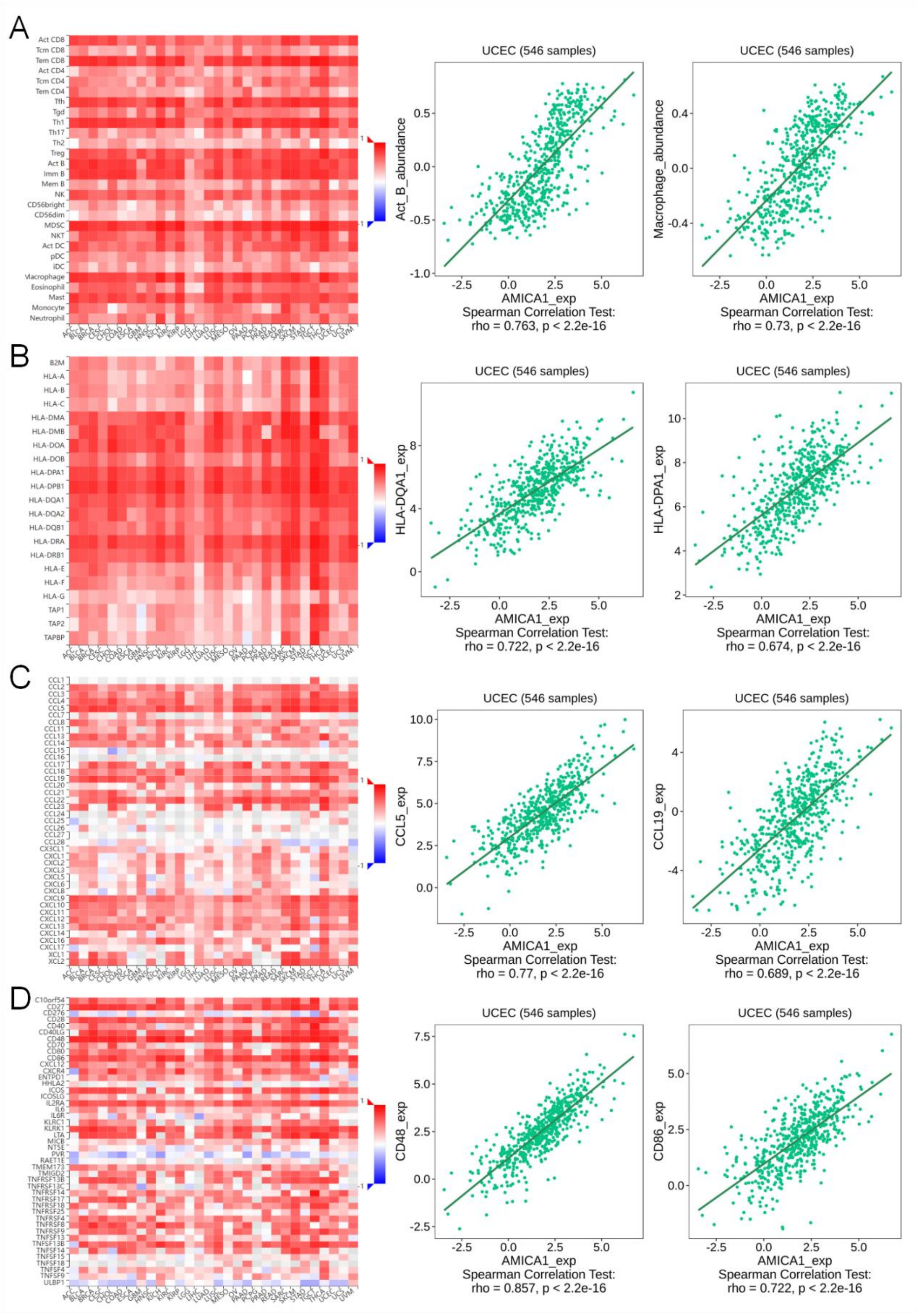

Supplementary Figure 3

Pan-cancer correlation analysis of JAML with immune features. For each analysis, the left panel shows a Spearman correlation heatmap across cancer types, and the right panel provides two representative scatter plots of specific correlations in UCEC. (A)

Spearman correlation between JAML expression and TILs across human cancers. (B) Spearman correlation between JAML expression and MHC molecules across human cancers. (C) Spearman correlation between JAML expression and chemokines across human cancers. (D) Spearman correlation between JAML expression and immune stimulatory factors across human cancers.

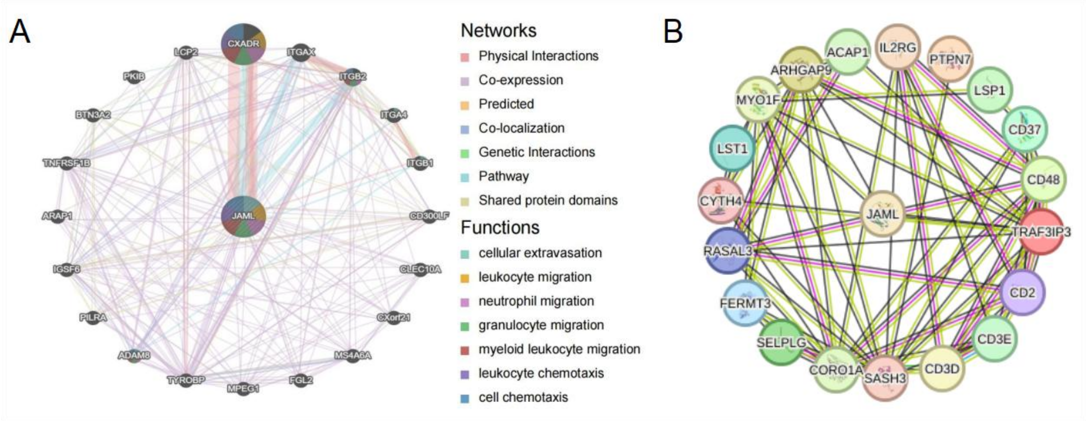

Supplementary Figure 4

JAML co-expression networks. (A) JAML co-expression network predicted by GeneMANIA. (B) Network of top 20 genes co-expressed with JAML in the UCEC cohort (TCGA, cBioPortal).

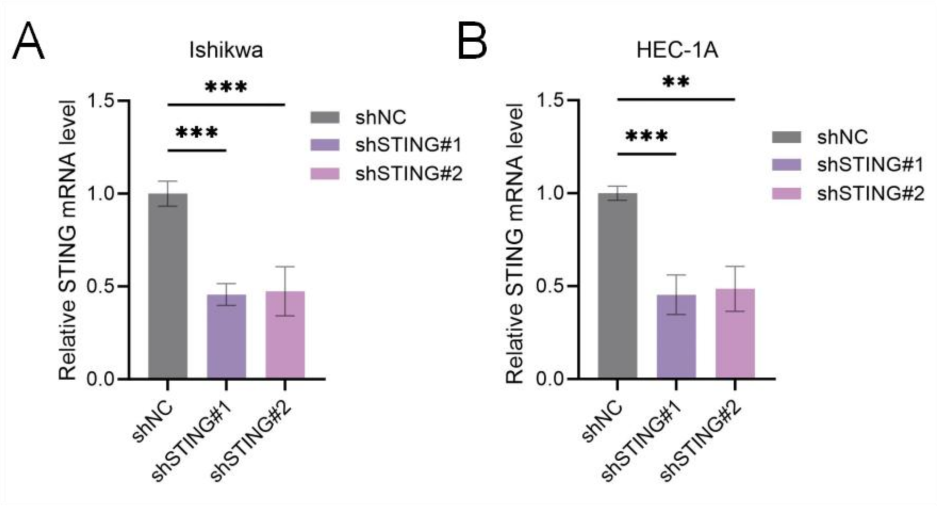

Supplementary Figure 5

STING knockdown efficiency validation. (A-B)Relative STING mRNA expression levels in Ishikawa and HEC-1A cells following transfection with shSTING. Note: Data are presented as mean ± SD. *p < 0.05, **p < 0.01, ***p < 0.001; ns, not significant.

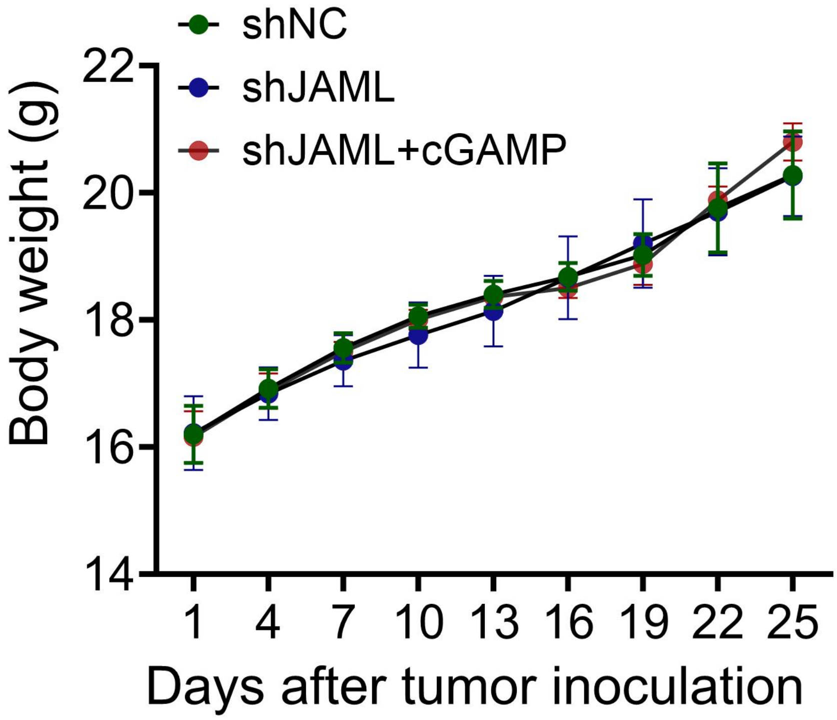

Supplementary Figure 6

Body weight monitoring during treatment. Mice were weighed every 3 days from tumor cell injection (Day 1). Data are mean ± SEM (n=5/group). No significant difference between groups was observed (two-way ANOVA, p>0.05).

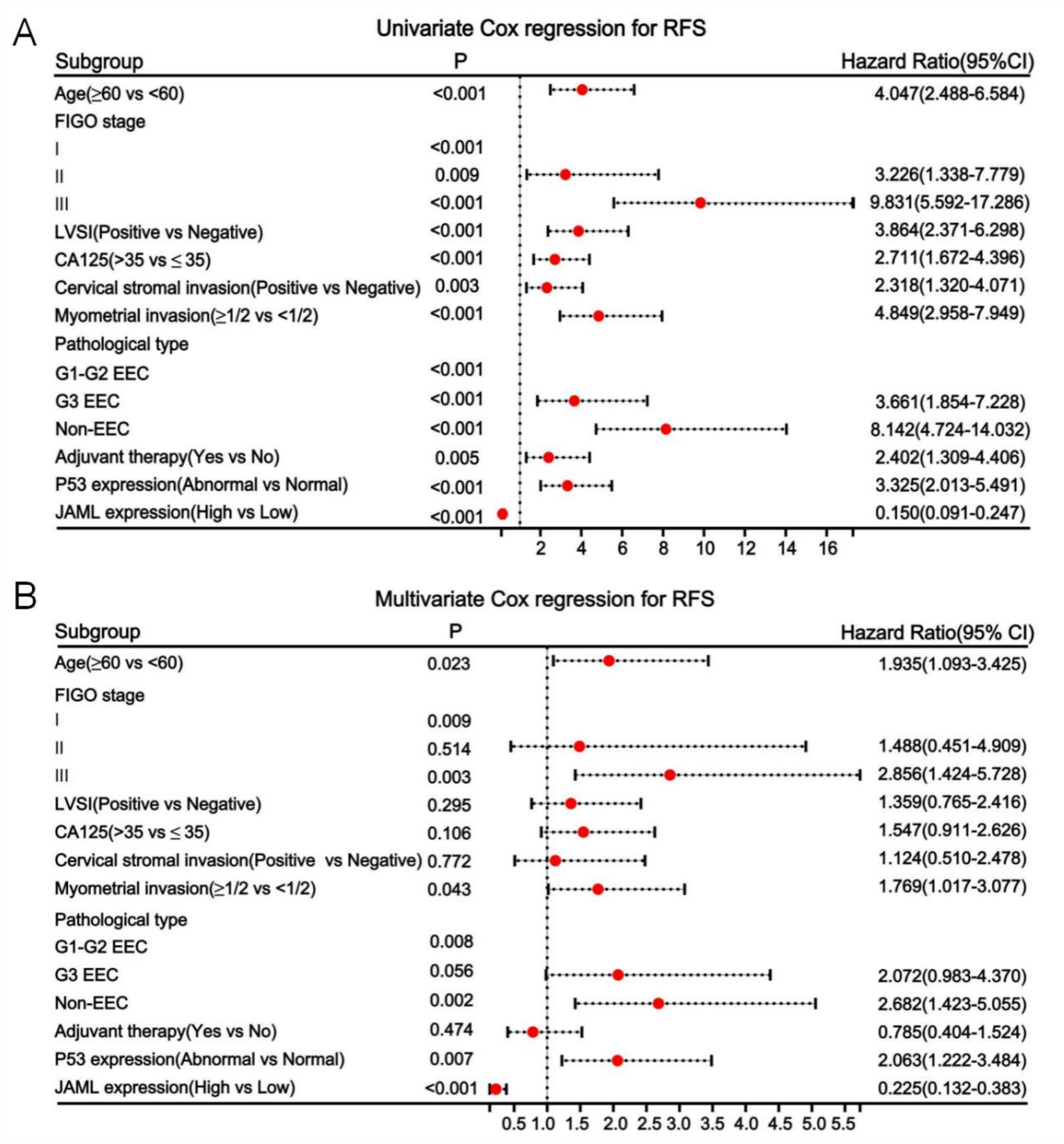

Supplementary Figure 7

Forest plots of Cox regression analyses for RFS in the training cohort. (A) Univariate Cox regression analysis of factors associated with RFS. (B) Multivariate Cox regression analysis of factors associated with RFS.

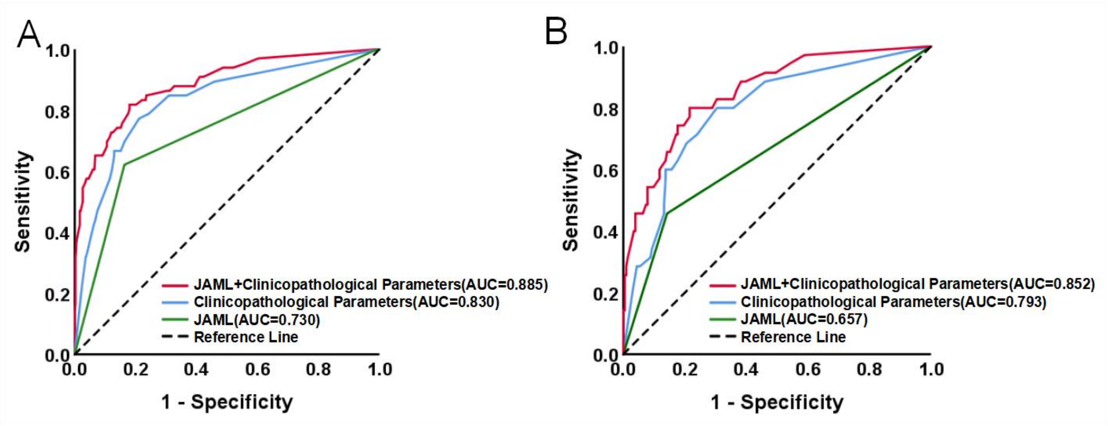

Supplementary Figure 8

Predictive performance of JAML, clinicopathological parameters, and their combination for RFS. (A) AUC values in the training cohort. (B) AUC values in the validation cohort.

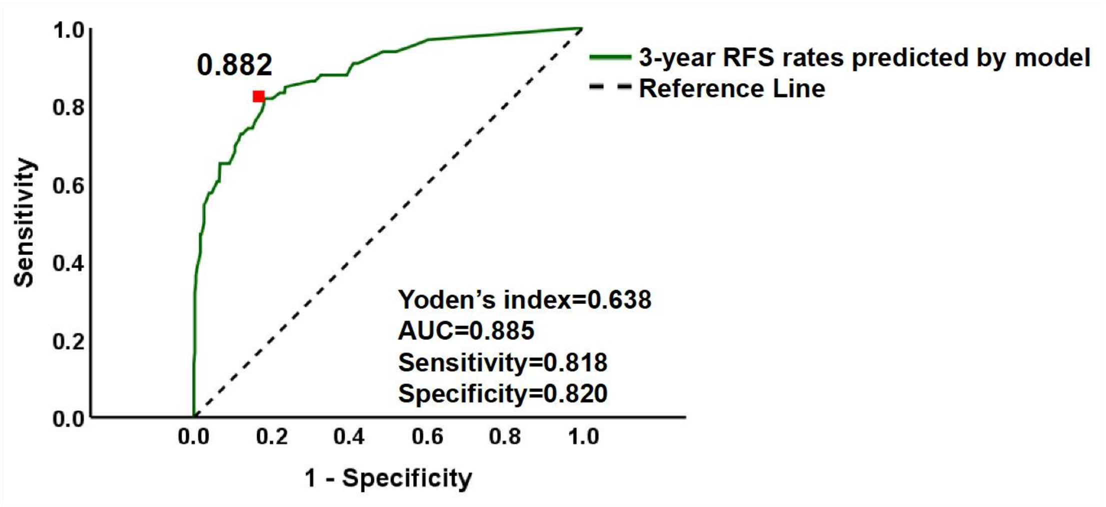

Supplementary Figure 9

ROC curve of the nomogram for predicting 3-year RFS in EC. The model achieved an AUC of 0.885, with a sensitivity of 0.818 and specificity of 0.820 at the optimal cut-off (0.882).

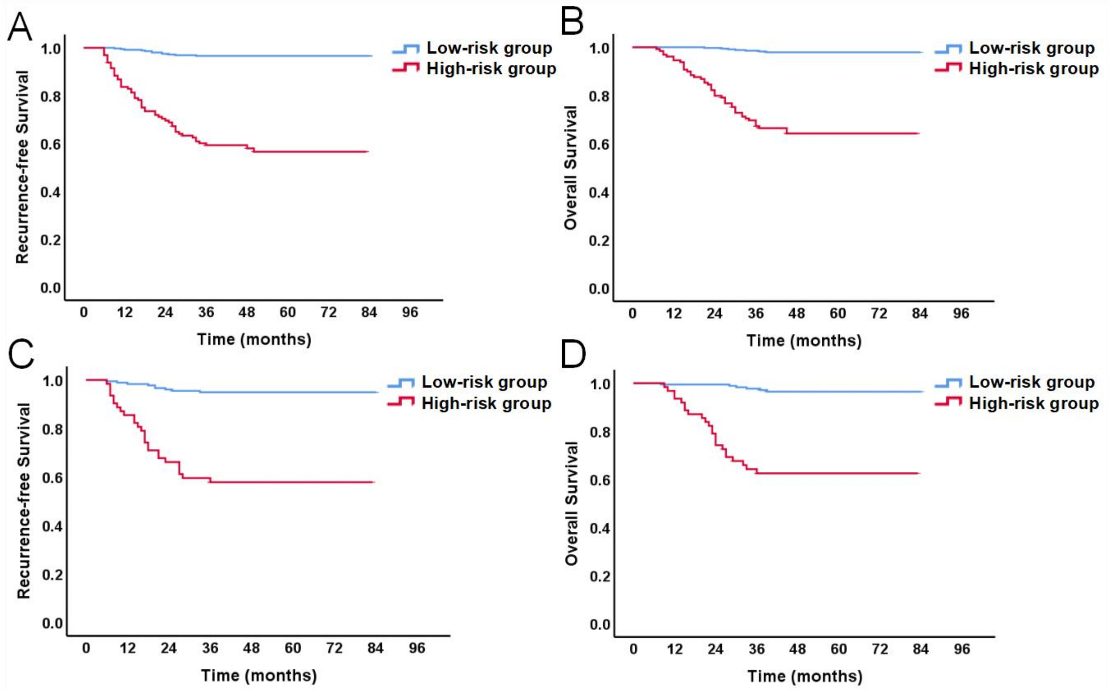

Supplementary Figure 10

Kaplan-Meier survival analysis based on the nomogram-defined risk groups. (A, B) RFS and OS in the training cohort. (C, D) RFS and OS in the validation cohort.
