# Supplementary material for: The prognostic significance of JAML and its role in remodeling the immune microenvironment via the cGAS-STING pathway in endometrial cancer
Source: Front Immunol. 2026 Jan 29;17:1738596. doi: 10.3389/fimmu.2026.1738596 (PMC12894416; doi:10.3389/fimmu.2026.1738596)
Supplement: Supplementary Table 1 — Bioinformatics tools and databases used in this study. [file Table1.docx]

**Supplementary Table 1 Bioinformatics tools and databases used in this study.**

| Databases | Abbreviation | URL |
| --- | --- | --- |
| Tumor Immune Estimation Resource | TIMER1.0 | https://cistrome.shinyapps.io/timer/ |
| Tumor-Immune System Interaction Database | TISIDB | http://cis.hku.hk/TISIDB/ |
| Human Protein Atlas | HPA | https://www.proteinatlas.org/ |
| Kaplan-Meier Plotter | KM Plotter | https://kmplot.com/analysis/ |
| Gene Multiple Association Network Integration Algorithm | GeneMANIA | https://genemania.org/ |
| cBioPortal for Cancer Genomics | cBioPortal | https://www.cbioportal.org/ |
| Metascape Gene List Analysis Portal | Metascape | http://metascape.org/ |
| Wei Sheng Xin | WSX | https://www.bioinformatics.com.cn/ |
| Genomics of Drug Sensitivity in Cancer | GDSC | https://www.cancerrxgene.org/ |
| STRING Protein-Protein Interaction Networks | STRING | https://string-db.org/ |

**Supplementary Table 2** **Inter-observer agreement for JAML expression by Cohen’s kappa in training and validation cohorts.**

| **Observer 2\ Observer 1** | JAML high | JAML low | **Total** | **Overall Agreement** | **Cohen’s κ (95% CI)** |
| --- | --- | --- | --- | --- | --- |
| **Training** (N=483) |  |  |  | 89.65% | 0.718 (0.608, 0.828) |
| JAML high | 341 | 33 | 374 |  |  |
| JAML low | 17 | 92 | 109 |  |  |
| Total | 358 | 125 | 483 |  |  |
| **Validation** (N=239) |  |  |  | 92.89% | 0.784 (0.686, 0.882) |
| JAML high | 181 | 11 | 192 |  |  |
| JAML low | 6 | 41 | 47 |  |  |
| **Total** | 187 | 52 | 239 |  |  |

**Note:** Cohen’s kappa coefficient was used to evaluate inter-observer agreement for dichotomized JAML expression (high vs. low). Agreement strength was interpreted as follows: κ < 0.20 (slight), 0.21–0.40 (fair), 0.41–0.60 (moderate), 0.61–0.80 (substantial), 0.81–1.00 (almost perfect).

**Supplementary Table 3 Inter-observer reliability of continuous immunohistochemistry assessments by ICC.**

| **Variable** | **N** | ****Single-Measures ICC****  ****(95% CI)**** | **Average-Measures ICC**  **(95% CI)** | ***P*** |
| --- | --- | --- | --- | --- |
| CD206^+^cells | 483 | 0.919 (0.904, 0.932) | 0.958 (0.949, 0.965) | ＜0.001 |
| CD86^+^cells | 483 | 0.902 (0.884, 0.918) | 0.949 (0.939, 0.957) | ＜0.001 |
| p-STING IHC score | 483 | 0.932 (0.920, 0.943) | 0.965 (0.958, 0.971) | ＜0.001 |

**Supplementary Table 4 Association between JAML expression and clinicopathological characteristics in the UCEC cohort.**

| **Characteristic** | **Total patients**  **(N=542)** | **High expression**  **(n=271)** | **Low expression**  **(n=271)** | ***P*** |
| --- | --- | --- | --- | --- |
| **Age, years**  <60  ≥60 | 179  363 | 100(36.9%)  171(63.1%) | 79(29.2%)  192(70.8%) | 0.055 |
| **FIGO stage**  I  II  III  IV | 337  52  124  29 | 185(68.3%)  19(7.0%)  58(21.4%)  9(3.3%) | 152(56.1%)  33(12.2%)  66(24.4%) 20(7.4%) | 0.009 |
| **Grade**  G1  G2  G3 High Grade | 99  121  311  11 | 61(22.5%)  68(25.1%)  138(50.9%)  4(1.5%) | 38(14.0%)  53(19.6%)  173(63.8%)  7(2.6%) | 0.008 |
| **Histological Subtypes**  EEC  Non-EEC | 406  136 | 220(81.2%)  51(18.8%) | 186(68.6%)  85(31.4%) | 0.001 |
| **TP53 Expression(n=528)**  Wild  Mutant | 331  197 | 182(68.9%)  82(31.1%) | 149(56.4%)  115(43.6%) | 0.003 |
| **Adjuvant therapy**  No Therapy  Radiotherapy  Chemotherapy  Chemo-radiotherapy | 237  71  105  129 | 124(45.8%)  26(9.6%)  60(22.1%)  61(22.5%) | 113(41.7%)  45(16.6%)  45(16.6%)  68(25.1%) | 0.044 |
| **Chemotherapy**  No  Yes | 342  200 | 184(67.9%)  87(32.1%) | 158(58.3%)  113(41.7%) | 0.021 |
| **Radiotherapy**  No  Yes | 308  234 | 150(55.4%)  121(44.6%) | 158(58.3%)  113(41.7%) | 0.488 |
| **Abbreviations:** FIGO, International Federation of Gynecology and Obstetrics; EEC, endometrioid endometrial carcinoma; EEC, endometrioid endometrial carcinoma;. | | | | |

**Note:** TP53 status data were available for 528 of the 542 patients, as indicated in the table.

**Supplementary Table 5 Association between JAML expression and clinicopathological characteristics of EC** **in the training cohort** **(N=483).**

| **Characteristic** | **High expression**  **(n=374)** | **Low expression (n=109)** | ***P*** |
| --- | --- | --- | --- |
| **Age, years**  <60  ≥60 | 282(75.4%)  92(24.6%) | 69(63.3%)  40(36.7%) | 0.013 |
| **BMI, kg/m^2^**  Mean ± SD | 24.35±3.75 | 24.59±3.44 | 0.554 |
| **FIGO stage**  I  II  III | 274(73.3%)  31(8.3%)  69(18.4%) | 60(55.0%)  12(11.0%)  37(33.9%) | 0.001 |
| **LVSI**  Negative  Positive | 286(76.5%)  88(23.5%) | 66(60.6%)  43(39.4%) | 0.001 |
| **CA125, U/mL**  ≤ 35  > 35 | 285(76.2%)  89(23.8%) | 71(65.1%)  38(34.9%) | 0.021 |
| **Cervical stromal invasion**  Negative  Positive | 330(88.2%)  44(11.8%) | 91(83.5%)  18(16.5%) | 0.192 |
| **Myometrial invasion**  <1/2  ≥1/2 | 288(77.0%)  86(23.0%) | 64(58.7%)  45(41.3%) | < 0.001 |
| **Pathological type**  G1-G2 EEC  G3 EEC  Non-EEC | 283(75.7%)  45(12.0%)  46(12.3%) | 70(64.2%)  15(13.8%)  24(22.0%) | 0.027 |
| **p53 expression**  Normal  Abnormal | 248(66.3%)  126(33.7%) | 60(55.0%)  49(45.0%) | 0.031 |
| **Adjuvant therapy**  No  Yes | 148(39.6%)  226(60.4%) | 25(22.9%)  84(77.1%) | 0.001 |
| **Abbreviations:** BMI, body mass index; FIGO, International Federation of Gynecology and Obstetrics; LVSI, lymphovascular space invasion; EEC, endometrioid carcinoma; SD, standard deviation. | | | |

**Supplementary Table 6 Comparison of Baseline Characteristics and Treatment Outcomes in Patients with Advanced or Recurrent EC Stratified by JAML Expression Levels (N=39).**

| **Variable** | **High expression**  **(n=12)** | **Low expression**  **(n=27)** | ***P*** |
| --- | --- | --- | --- |
| **Age, years**  mean ± SD | 57.67 ± 7.98 | 56.67 ± 9.53 | 0.753 |
| **BMI, kg/m^2^**  mean ± SD | 23.46 ± 4.14 | 23.23 ± 2.76 | 0.834 |
| **FIGO Stage**  III  IV | 9 (75.0%)  3 (25.0%) | 14 (51.9%)  13 (48.1%) | 0.158 |
| **Pathological type**  G1-G2 EEC  G3 EEC  Non-EEC | 5 (41.7%)  6 (50.0%)  1 (8.3%) | 8 (29.6%)  13 (48.1%)  6 (22.2%) | 0.530 |
| **ORR**  Objective Responder  Non-responder | 8 (66.7%)  4 (33.3%) | 7 (25.9%)  20 (74.1%) | 0.020 |
| **PFS, months**  Median [P25, P75] | 31.00 [29.25,39.50] | 11.00 [5.00, 30.00] | 0.027 |
| **Abbreviations:** BMI, body mass index; EEC, Endometrioid Endometrial Carcinoma; FIGO, International Federation of Gynecology and Obstetrics; ICI, Immune Checkpoint Inhibitor; ORR, Objective Response Rate; P25, 25th Percentile; P75, 75th Percentile; PFS, Progression-Free Survival; SD, Standard Deviation | | | |

**Supplementary Table 7** **Clinicopathological Characteristics of Patients with EC in the Training and Validation Cohorts.**

| **Characteristic** | **Training Cohort**  **(n=483)** | **Validation Cohort**  **(n=239)** | ***P*** |
| --- | --- | --- | --- |
| **Age, years**  mean ± SD | 53.99 ± 9.39 | 54.73 ± 8.69 | 0.306 |
| **BMI, kg/m^2^**  mean ± SD | 24.41±3.68 | 24.47±3.70 | 0.823 |
| **FIGO stage**  I  II  III | 334  43  106 | 167  20  52 | 0.967 |
| **LVSI**  Negative  Positive | 347  136 | 174  65 | 0.786 |
| **CA125(U/ml)**  ≤ 35  > 35 | 356  127 | 180  59 | 0.642 |
| **Cervical stromal invasion**  Negative  Positive | 421  62 | 210  29 | 0.789 |
| **Myometrial invasion**  <1/2  ≥1/2 | 352  131 | 165  74 | 0.282 |
| **Pathological type**  G1-G2 EEC  G3 EEC  Non-EEC | 353  60  70 | 171  28  40 | 0.724 |
| **P53 expression**  Normal  Abnormal | 308  175 | 152  87 | 0.964 |
| **Adjuvant therapy**  No  Yes | 173  310 | 82  157 | 0.690 |
| **Abbreviations:** BMI, body mass index; FIGO, International Federation of Gynecology and Obstetrics; LVSI, lymphovascular space invasion; EEC, endometrioid carcinoma; SD, standard deviation. | | | |
