# Supplementary material for: The prognostic significance of JAML and its role in remodeling the immune microenvironment via the cGAS-STING pathway in endometrial cancer
Source: Front Immunol. 2026 Jan 29;17:1738596. doi: 10.3389/fimmu.2026.1738596 (PMC12894416; doi:10.3389/fimmu.2026.1738596)

The original western-blot images

Figure 1J

Figure 1J——JAML（AMICA1）

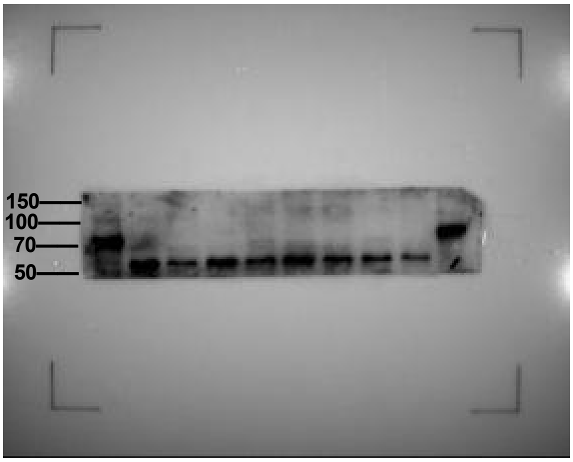

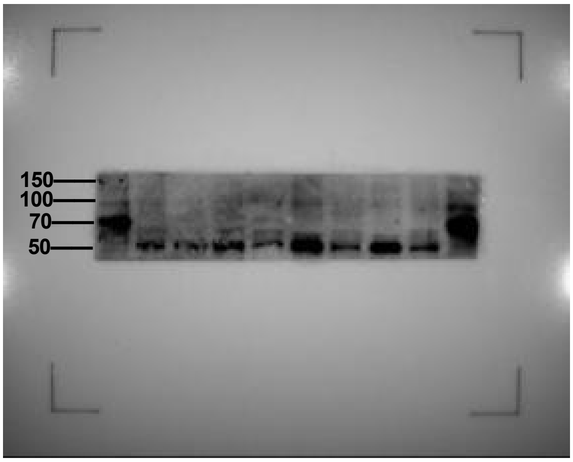

Figure 1J——GAPDH

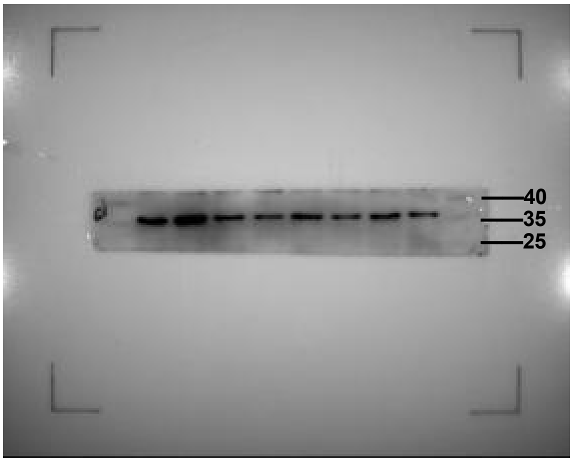

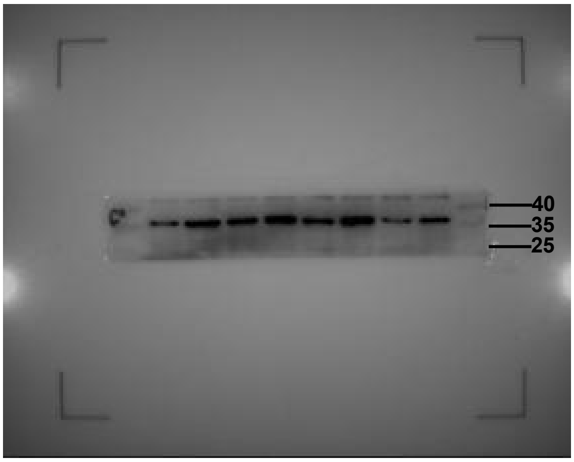

Figure 2A

Figure 2A: Ishikawa_JAML

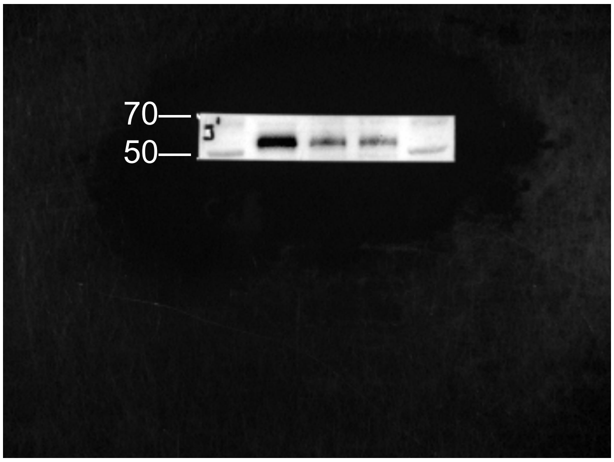

Figure 2A: Ishikawa_JAML

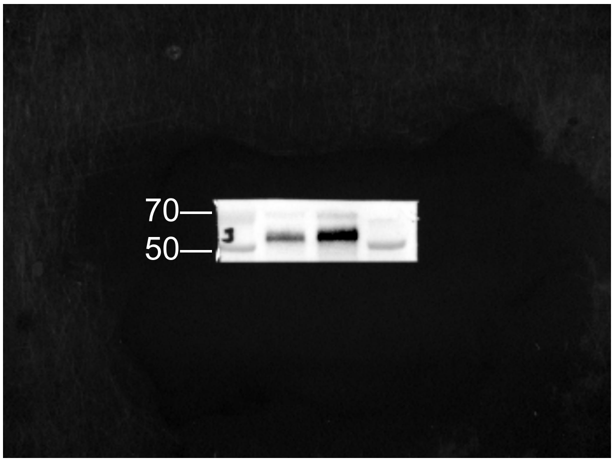

Figure 2A: Ishikawa_Ki67

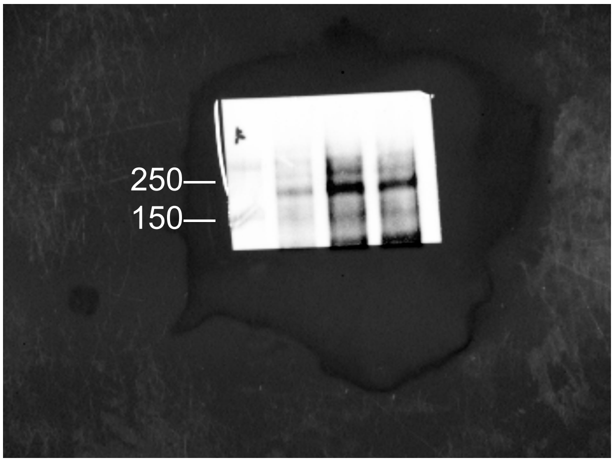

Figure 2A: Ishikawa_Ki67

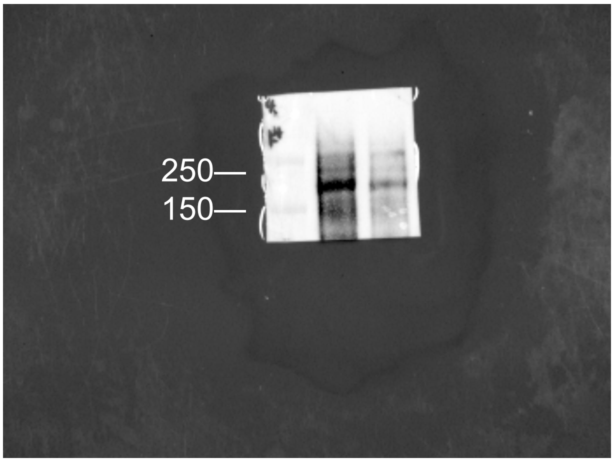

Figure 2A: Ishikawa_Vimentin

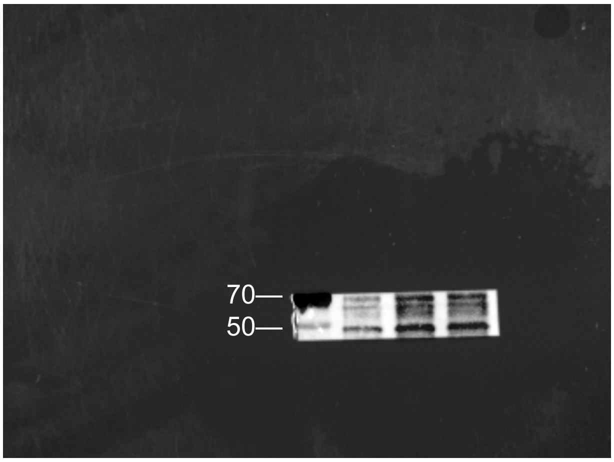

Figure 2A: Ishikawa_Vimentin

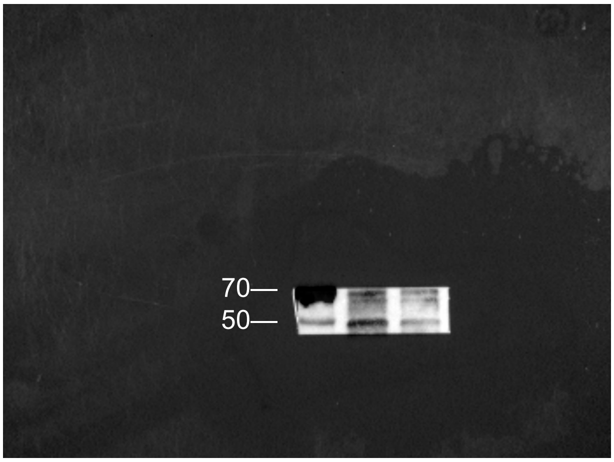

Figure 2A

Figure 2A: Ishikawa_N-cadherin

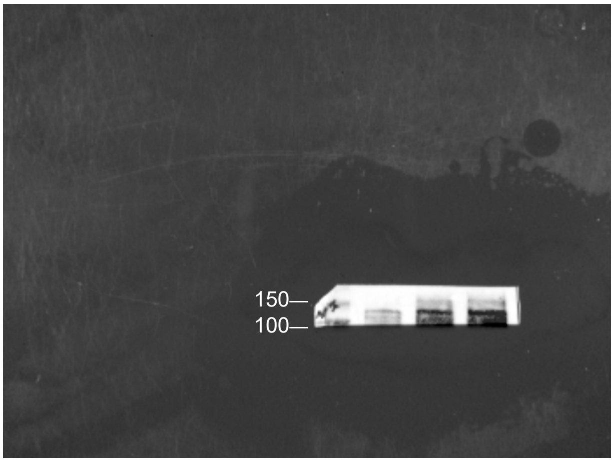

Figure 2A: Ishikawa_N-cadherin

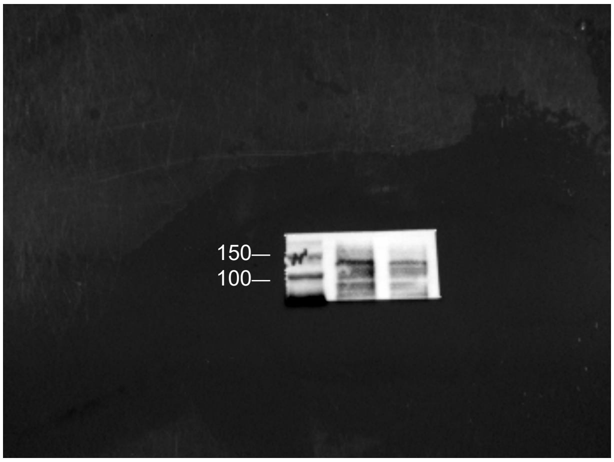

Figure 2A: Ishikawa_E-cadherin

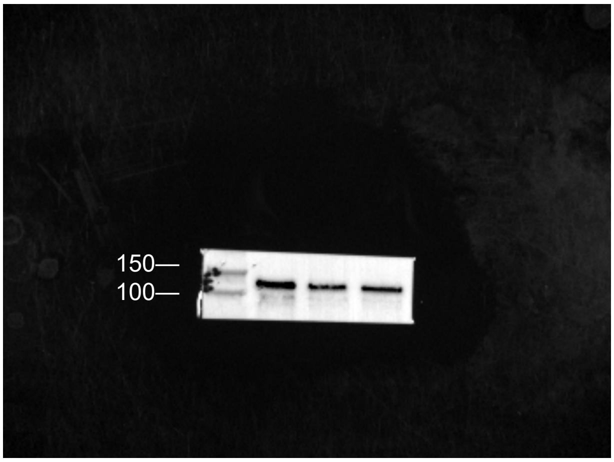

Figure 2A: Ishikawa_E-cadherin

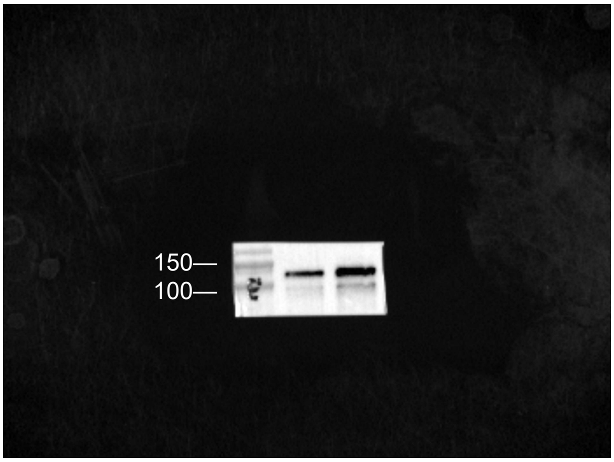

Figure 2A: Ishikawa_GAPDH

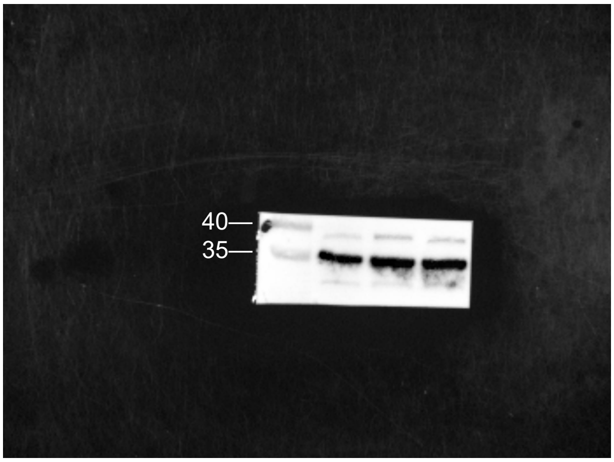

Figure 2A: Ishikawa_GAPDH

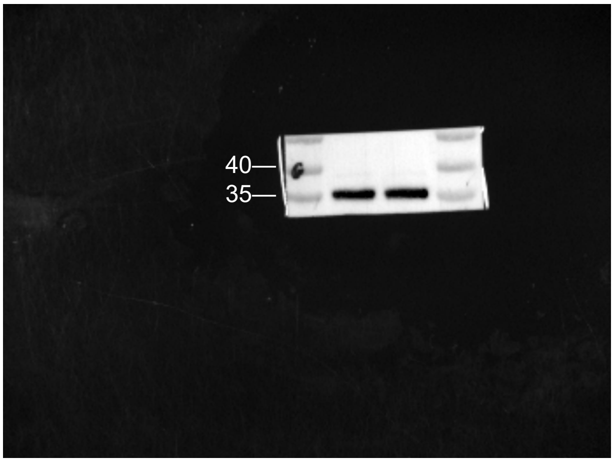

Figure 2B

Figure 2B: HEC-1A_JAML

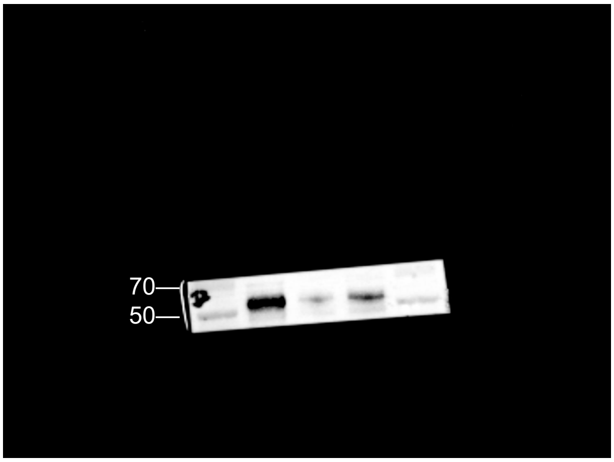

Figure 2B: HEC-1A_JAML

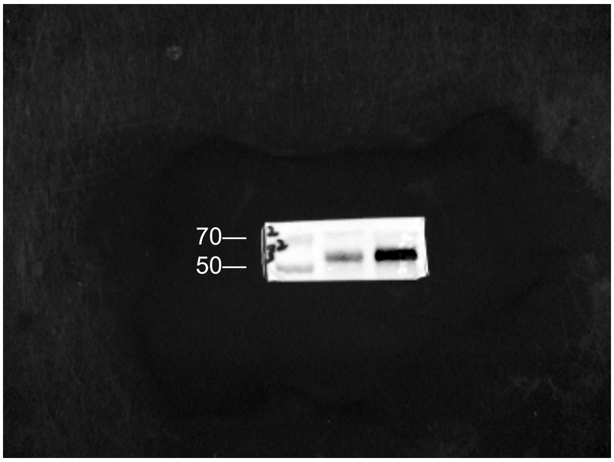

Figure 2B: HEC-1A_Ki67

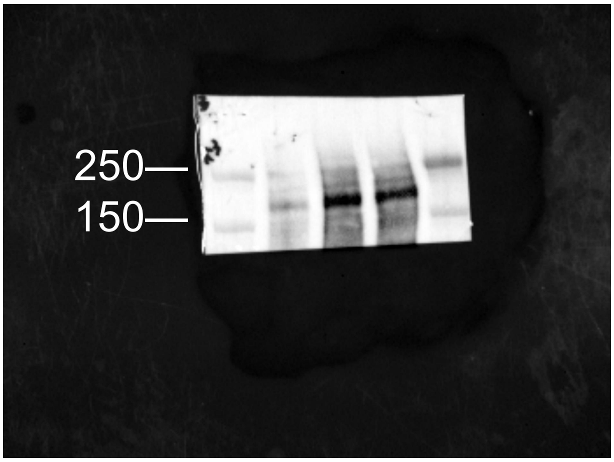

Figure 2B: HEC-1A_Ki67

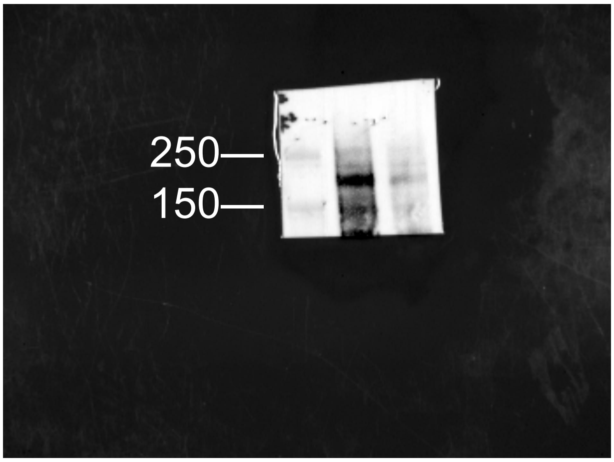

Figure 2B: HEC-1A_Vimentin

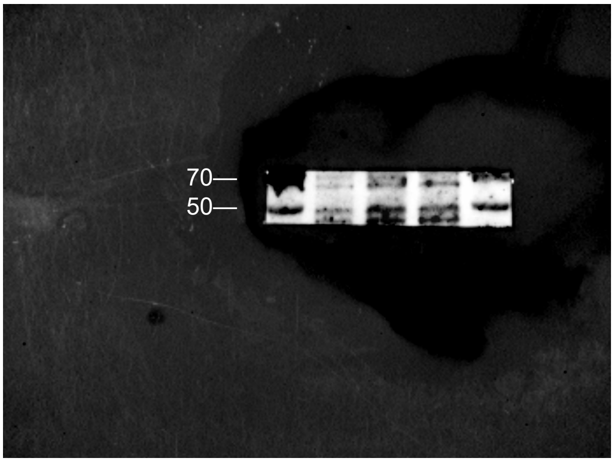

Figure 2B: HEC-1A_Vimentin

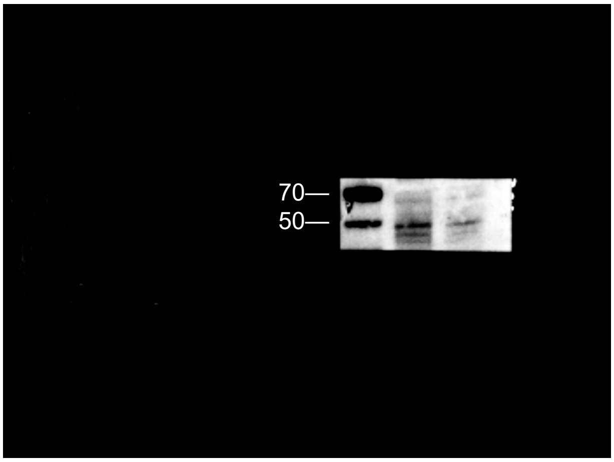

Figure 2B

Figure 2B: HEC-1A_N-cadherin

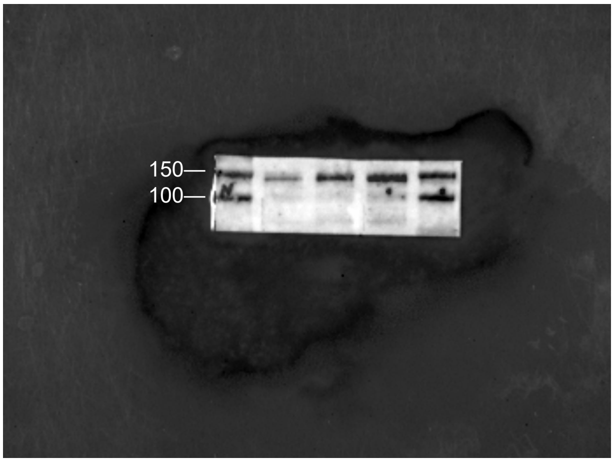

Figure 2B: HEC-1A_N-cadherin

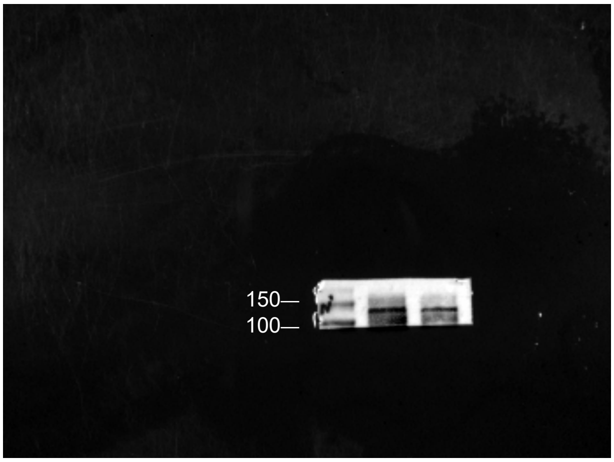

Figure 2B: HEC-1A_E-cadherin

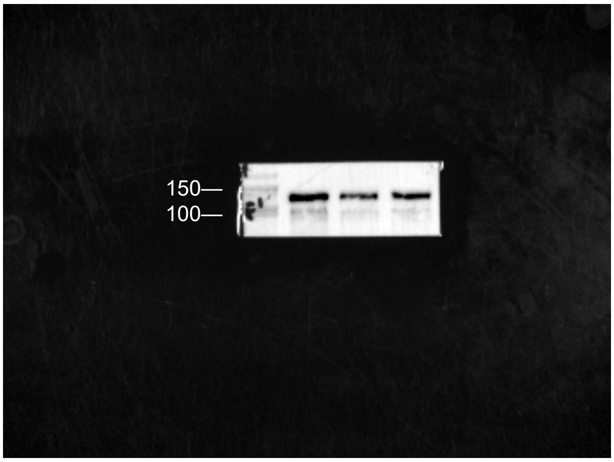

Figure 2B: HEC-1A_E-cadherin

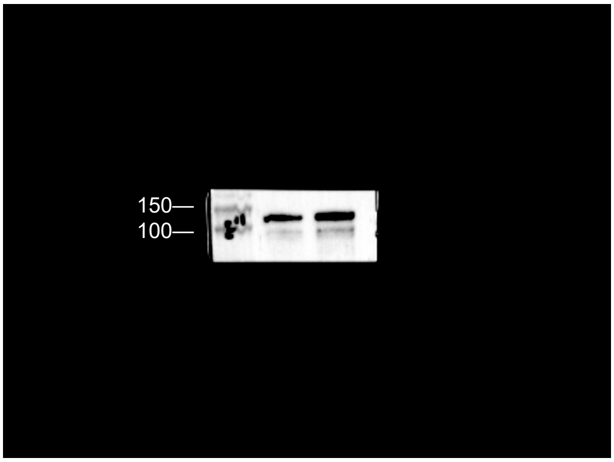

Figure 2B: HEC-1A_GAPDH

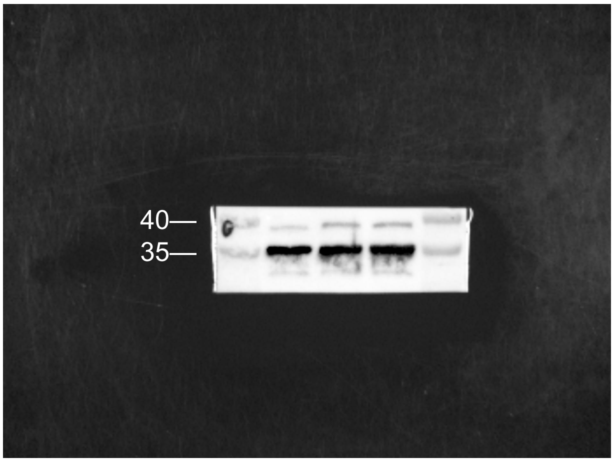

Figure 2B: HEC-1A_GAPDH

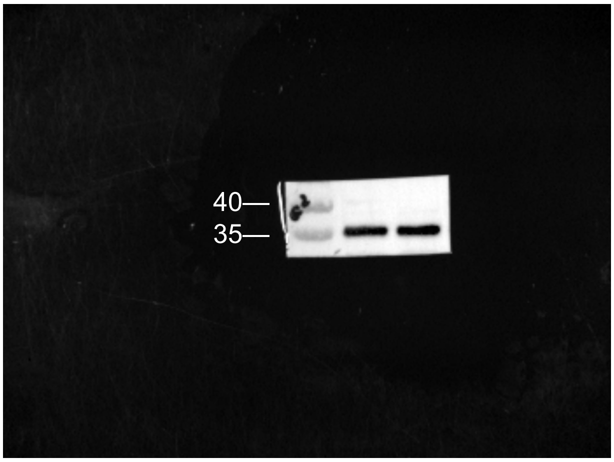

Figure 4C

Figure 4C: Ishikawa_JAML

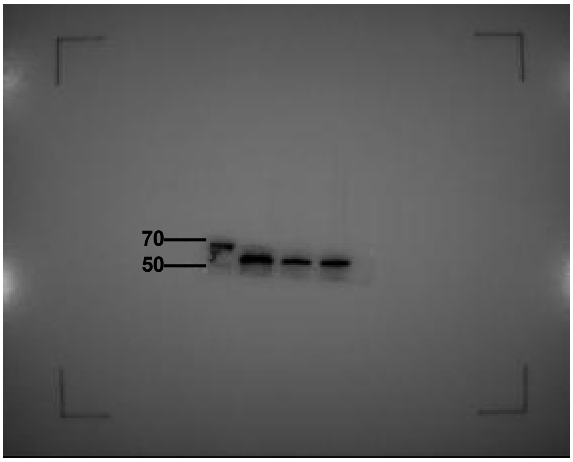

Figure 4C: Ishikawa_JAML

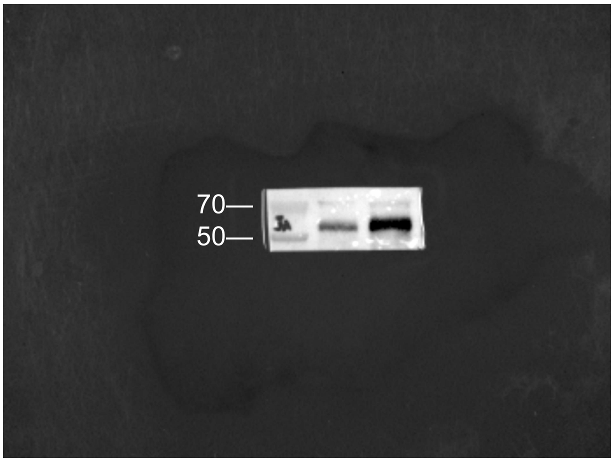

Figure 4C: Ishikawa_cGAS

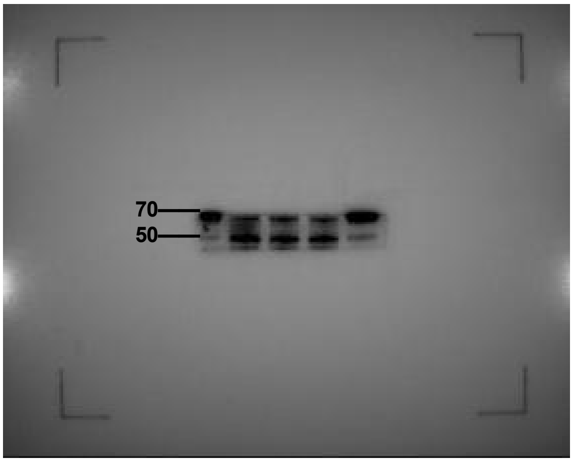

Figure 4C: Ishikawa_cGAS

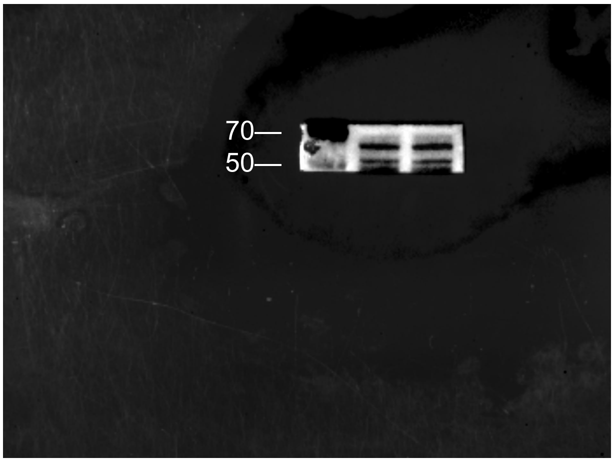

Figure 4C: Ishikawa_STING

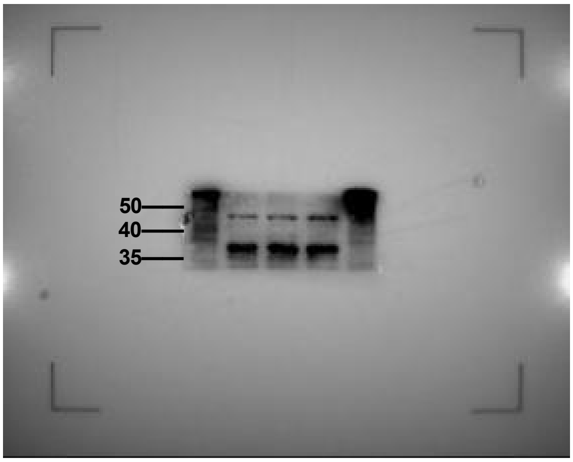

Figure 4C: Ishikawa_STING

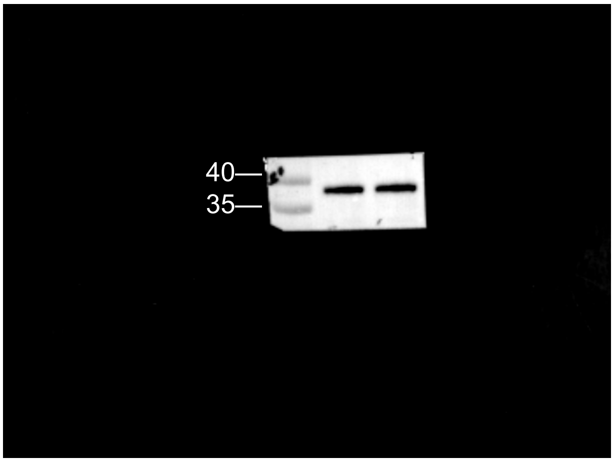

Figure 4C

Figure 4C: Ishikawa_p-STING

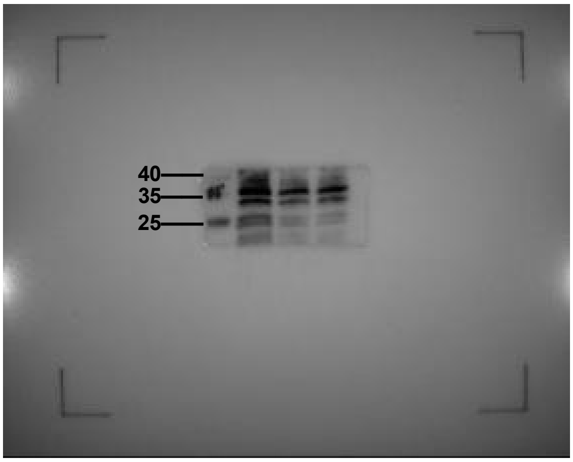

Figure 4C: Ishikawa_p-STING

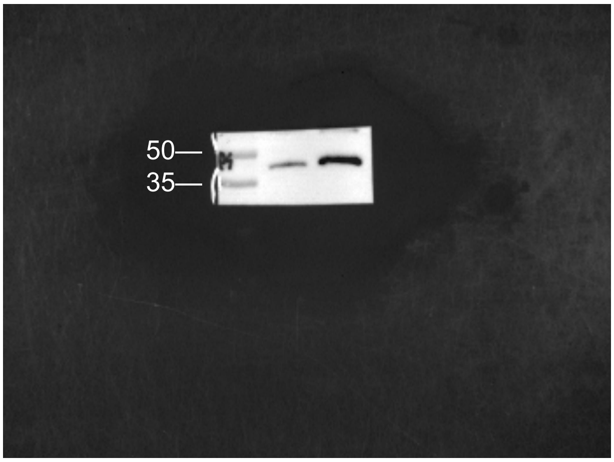

Figure 4C: Ishikawa_TBK1

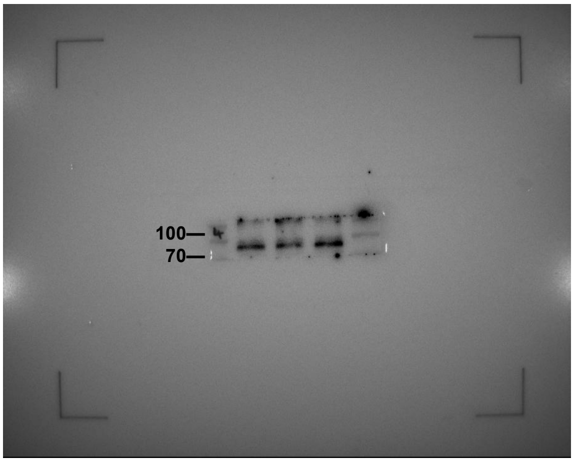

Figure 4C: Ishikawa_TBK1

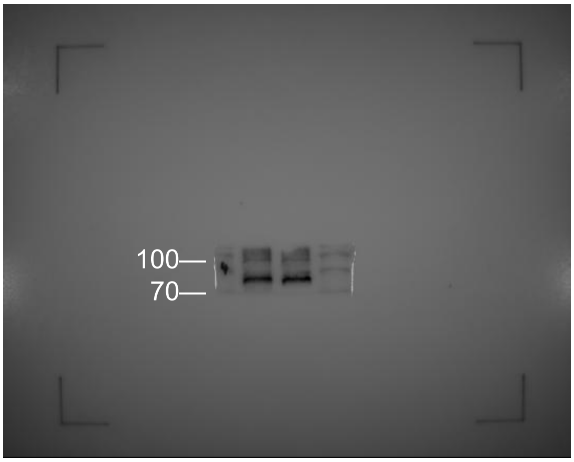

Figure 4C: Ishikawa_p-TBK1

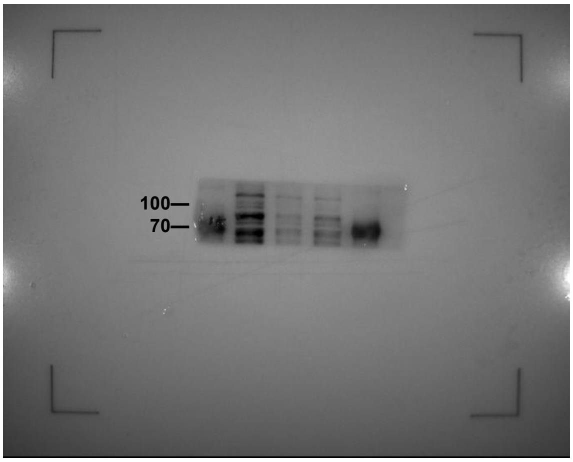

Figure 4C: Ishikawa_p-TBK1

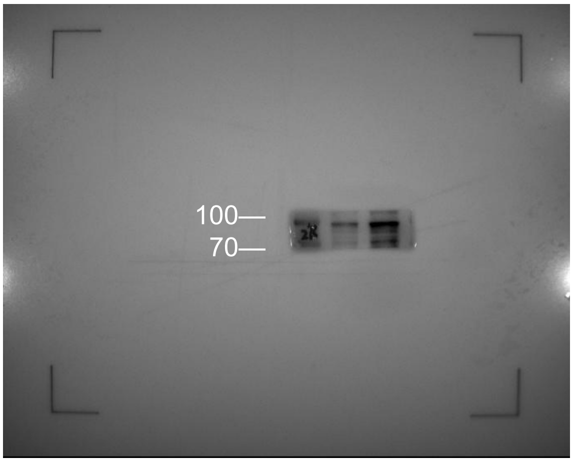

Figure 4C

Figure 4C: Ishikawa_IRF3

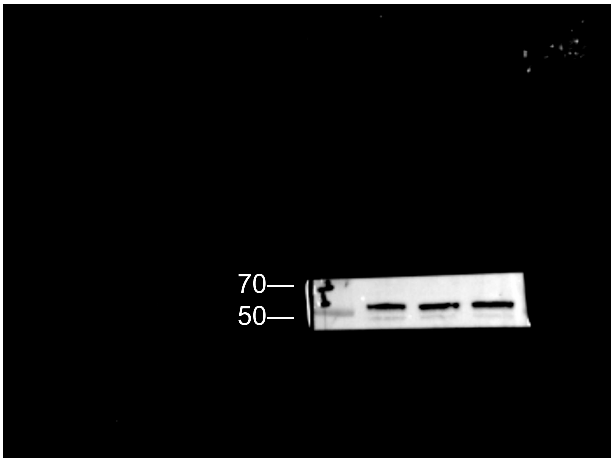

Figure 4C: Ishikawa_IRF3

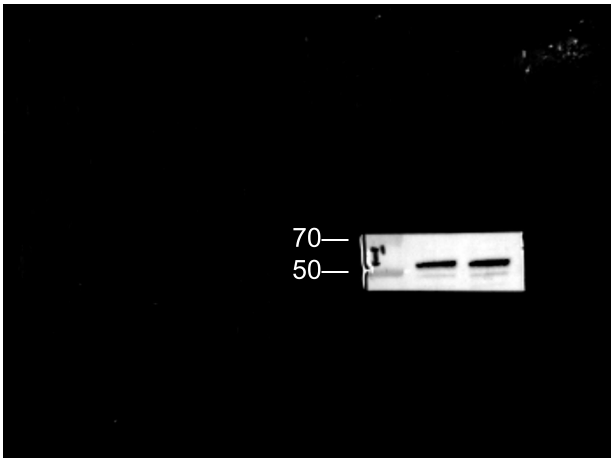

Figure 4D: Ishikawa_p-IRF3

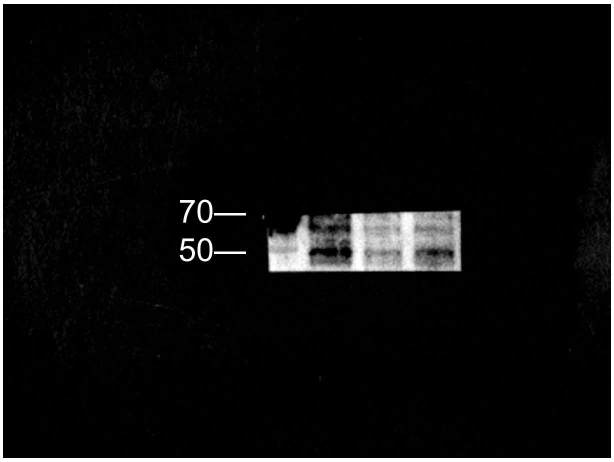

Figure 4C: Ishikawa_p-IRF3

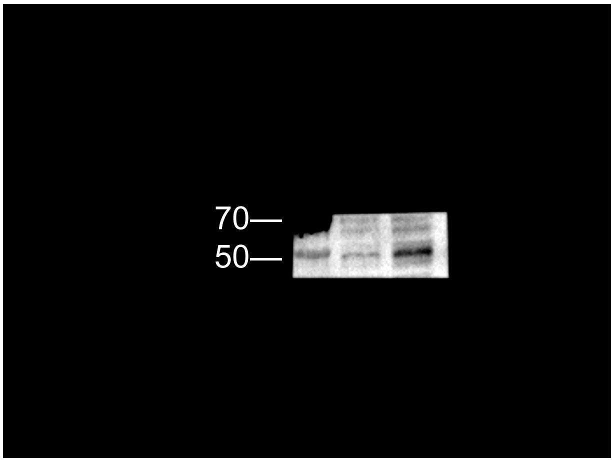

Figure 4C: Ishikawa_GAPDH

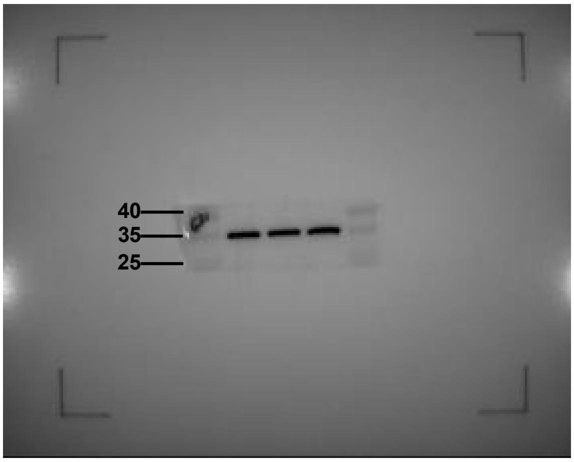

Figure 4C: Ishikawa_GAPDH

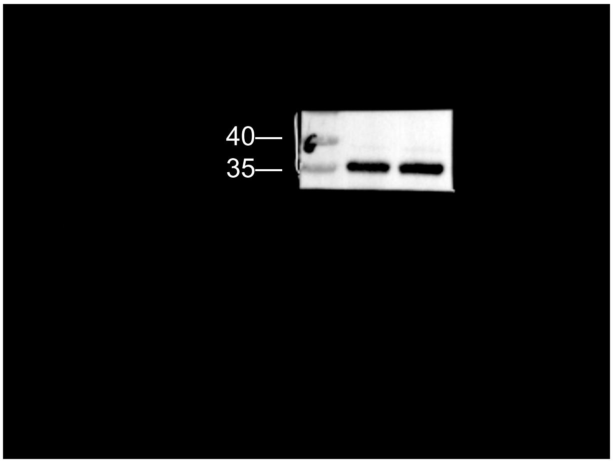

Figure 4D

Figure 4D: HEC-1A_JAML

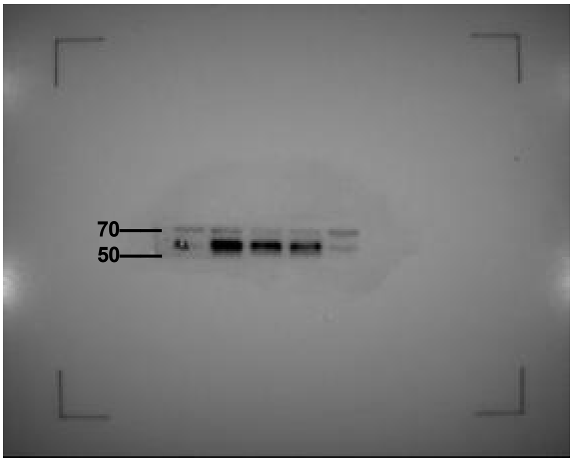

Figure 4D: HEC-1A_JAML

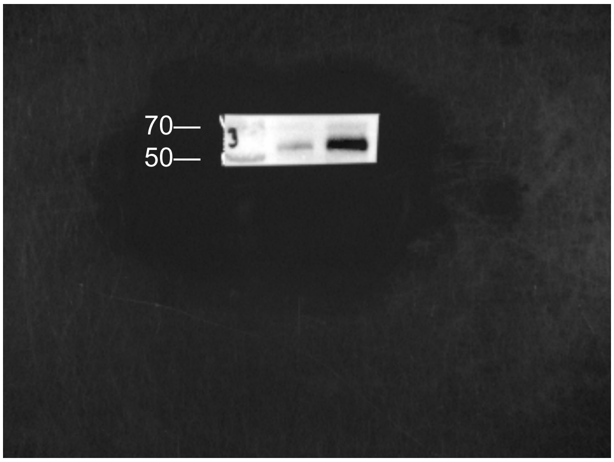

Figure 4D: HEC-1A_cGAS

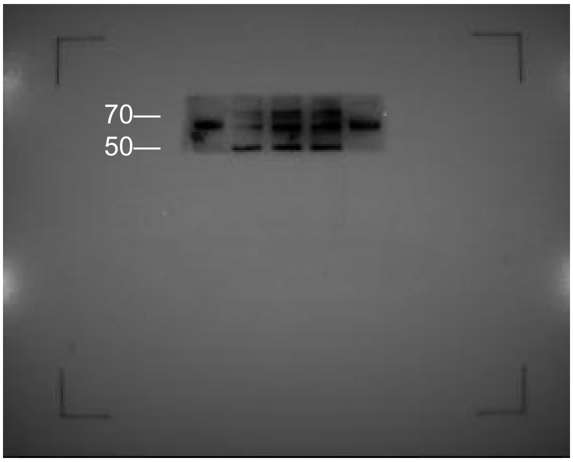

Figure 4D: HEC-1A_cGAS

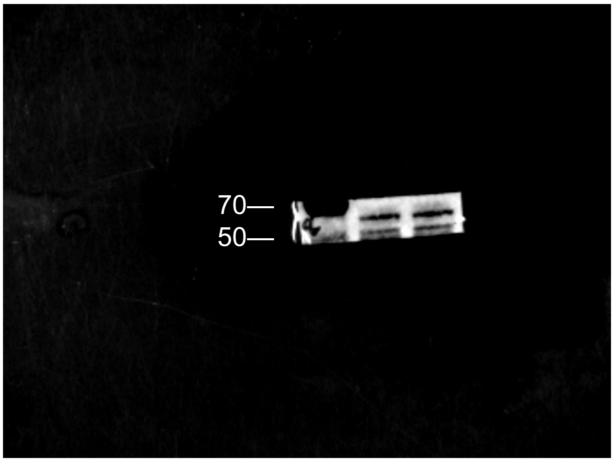

Figure 4D: HEC-1A_STING

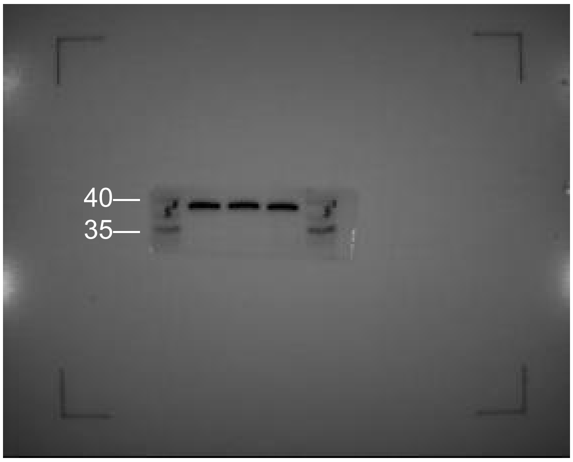

Figure 4D: HEC-1A_STING

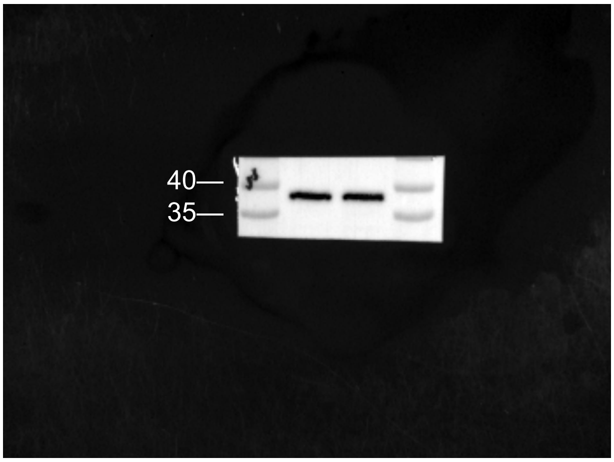

Figure 4D

Figure 4D: HEC-1A_p-STING

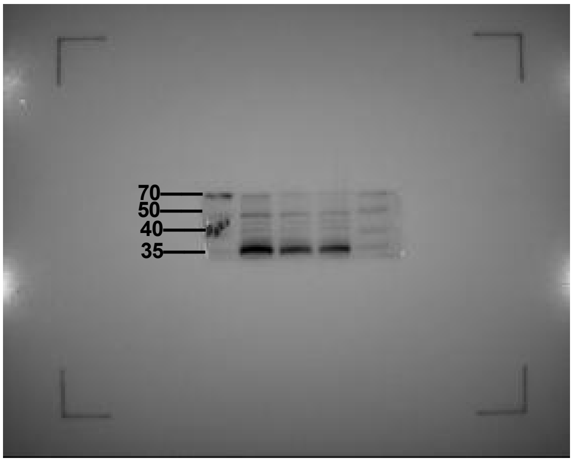

Figure 4D: HEC-1A_p-STING

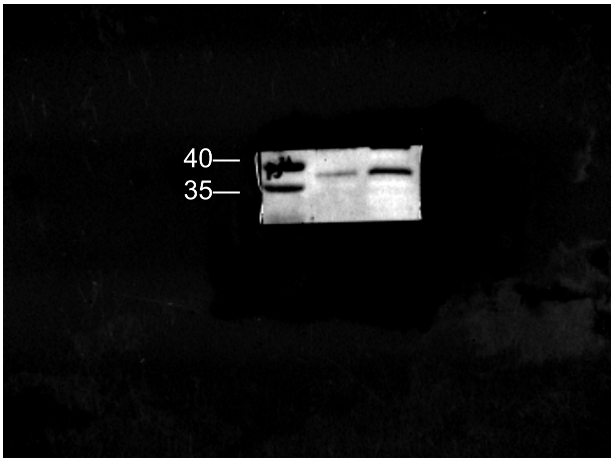

Figure 4D: HEC-1A_TBK1

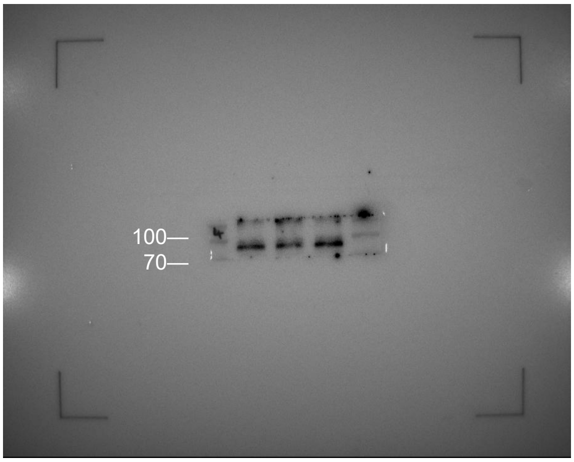

Figure 4D: HEC-1A_TBK1

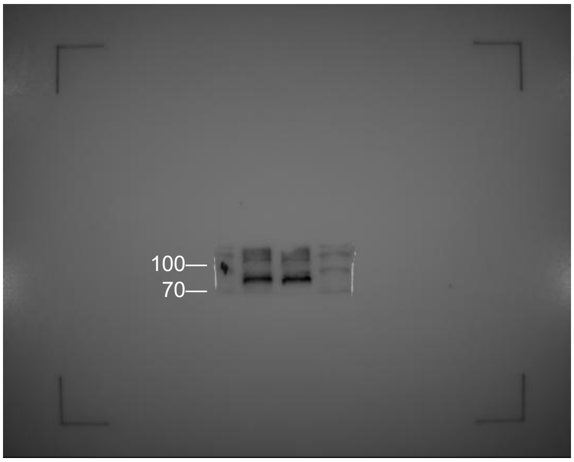

Figure 4D: HEC-1A_p-TBK1

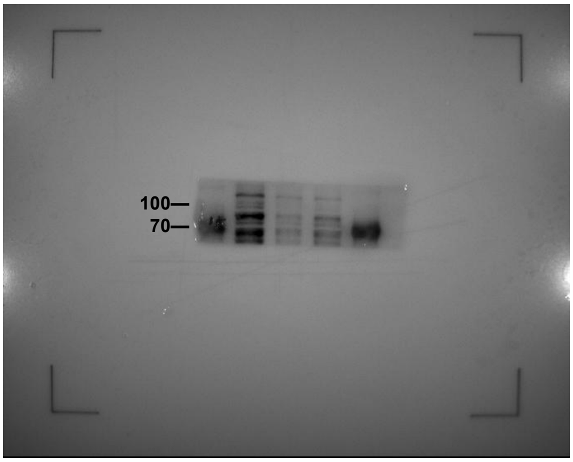

Figure 4D: HEC-1A_p-TBK1

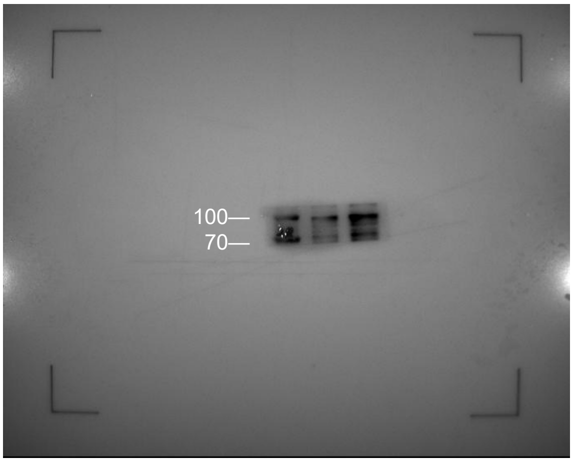

Figure 4D

Figure 4D:HEC-1A_IRF3

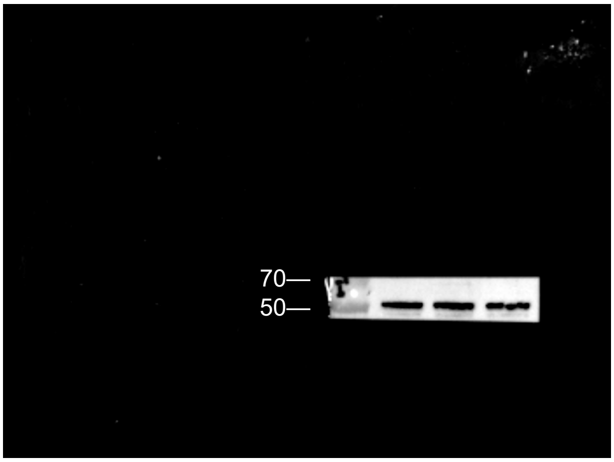

Figure 4D: HEC-1A_IRF3

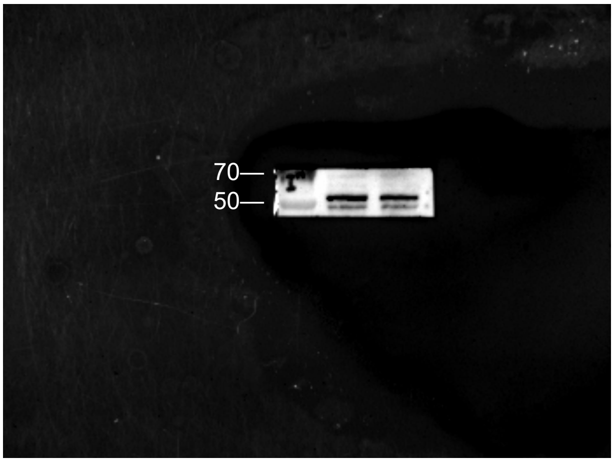

Figure 4D: HEC-1A_p-IRF3

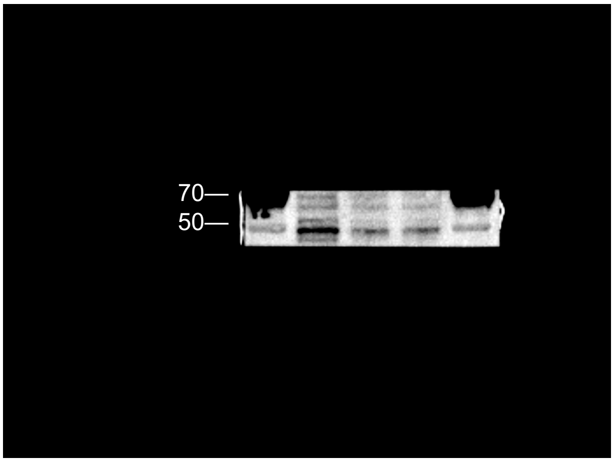

Figure 4D: HEC-1A_p-IRF3

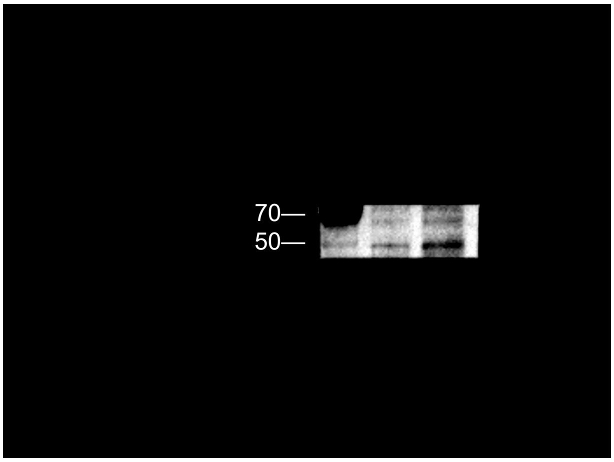

Figure 4D: HEC-1A_GAPDH

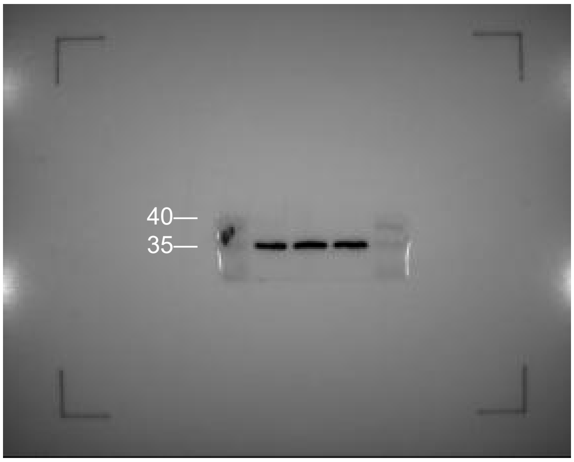

Figure 4D: HEC-1A_GAPDH

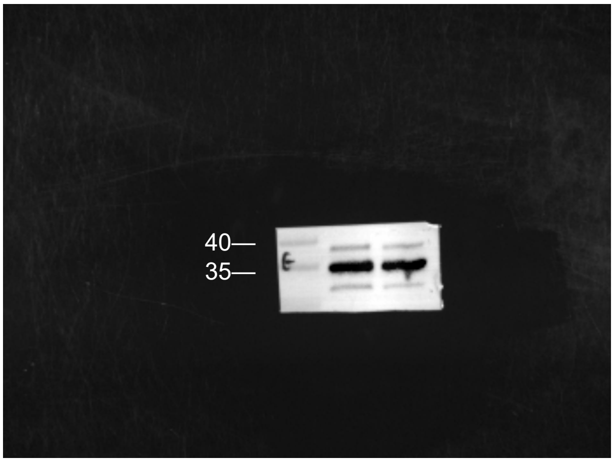

Figure 4E

Figure 4E:Ishikawa_JAML

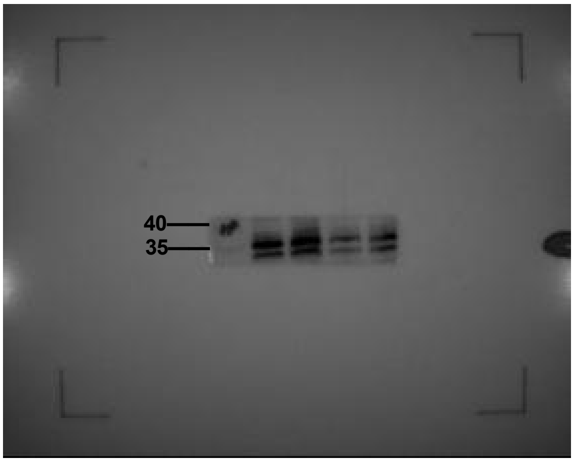

Figure 4E:HEC-1A_JAML

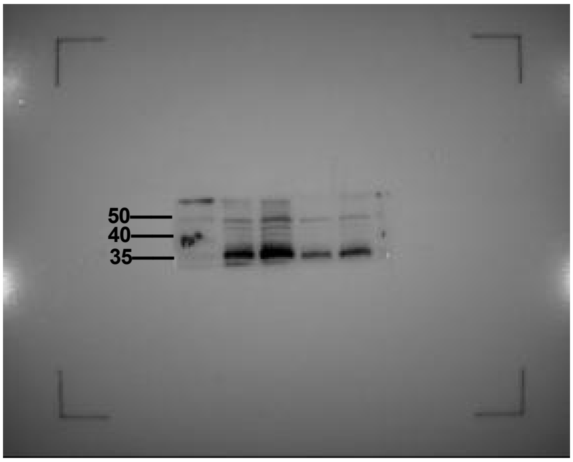

Figure 4E:Ishikawa_cGAS

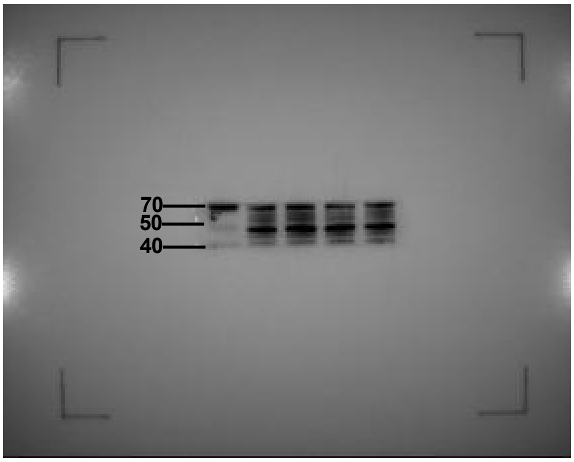

Figure 4E: HEC-1A_cGAS

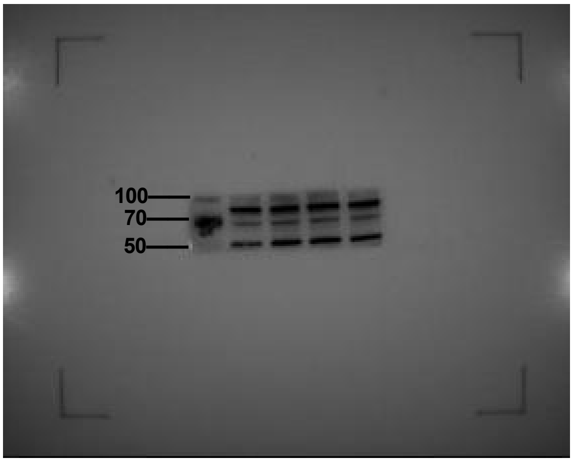

Figure 4E:Ishikawa_STING

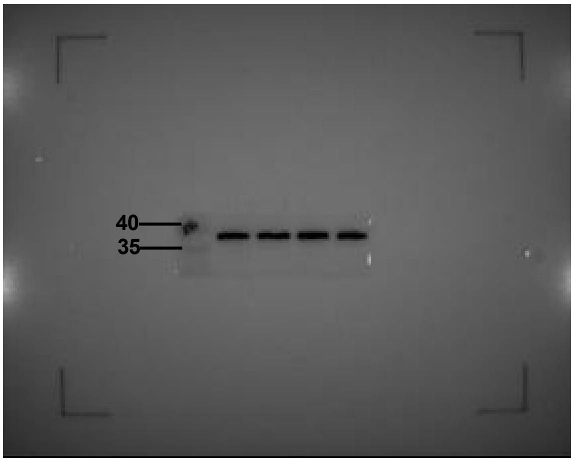

Figure 4E:HEC-1A_STING

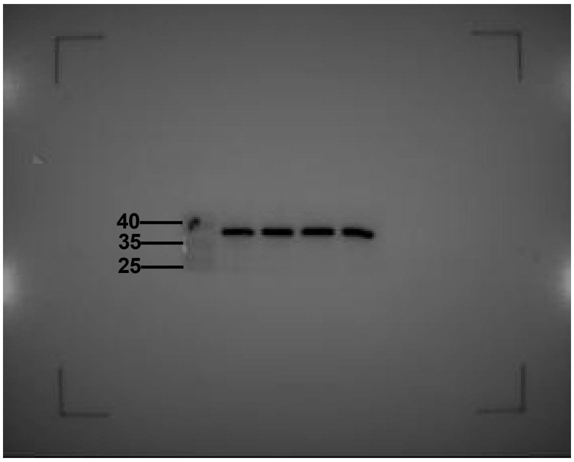

Figure 4E

Figure 4E:Ishikawa_p-STING

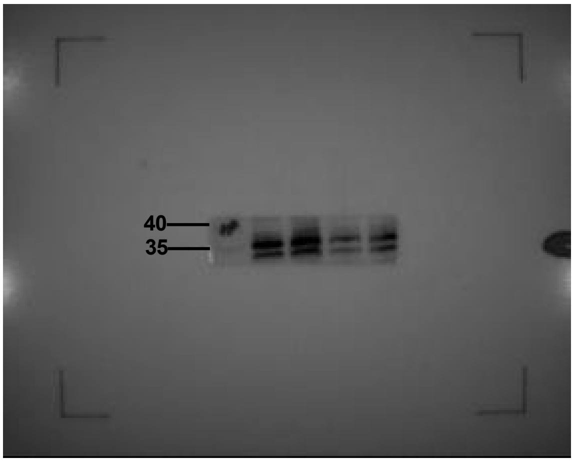

Figure 4E:HEC-1A_p-STING

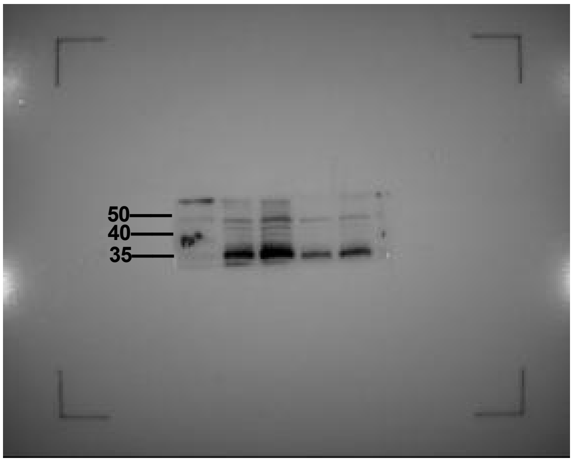

Figure 4E:Ishikawa_TBK1

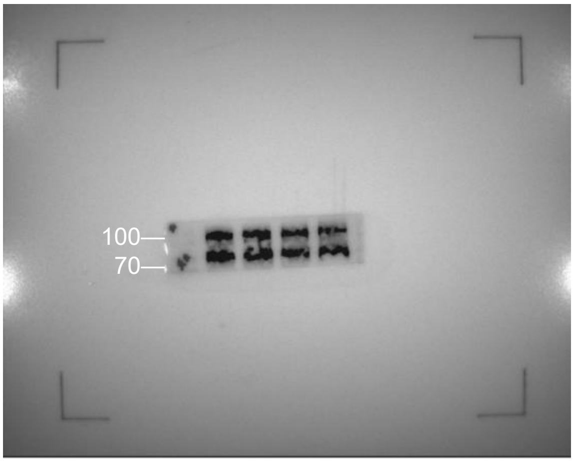

Figure 4E:HEC-1A_TBK1

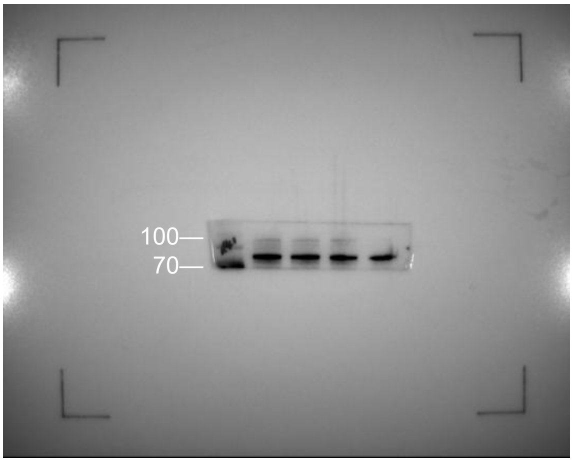

Figure 4E:Ishikawa_p-TBK1

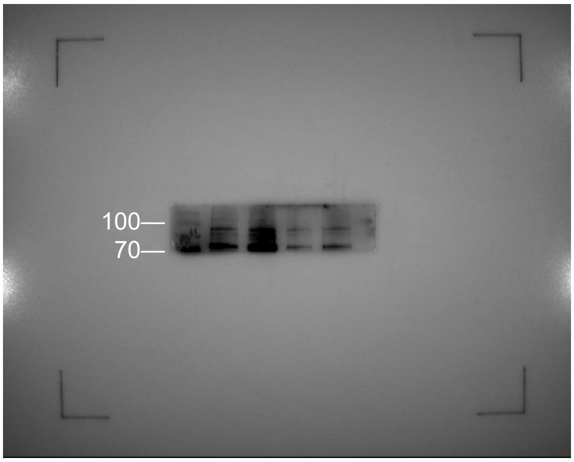

Figure 4F:HEC-1A_p-TBK1

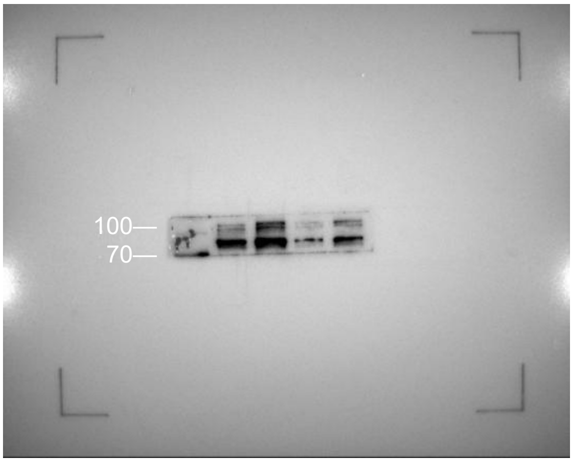

Figure 4E

Figure 4E:Ishikawa_IRF3

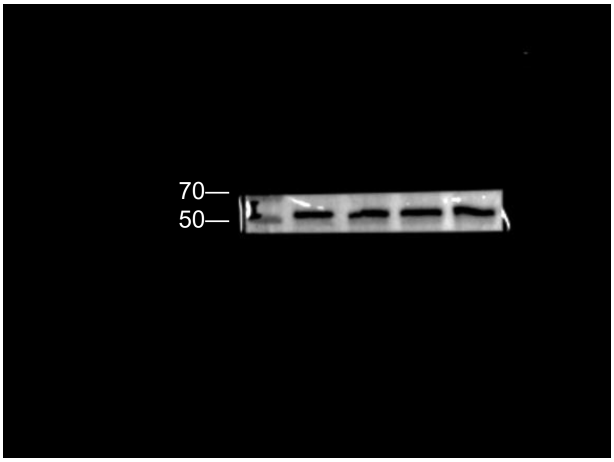

Figure 4E: HEC-1A_IRF3

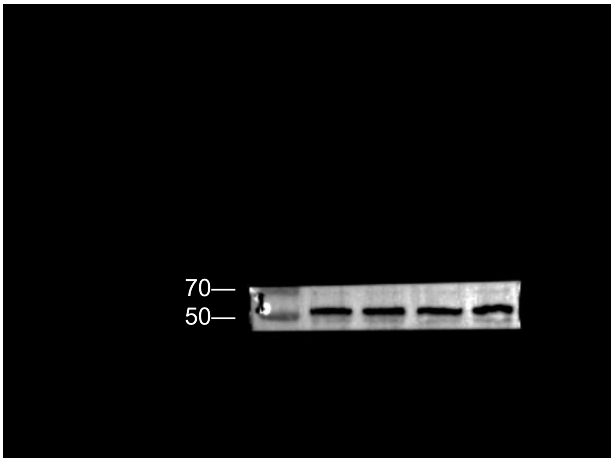

Figure 4E:Ishikawa_p-IRF3

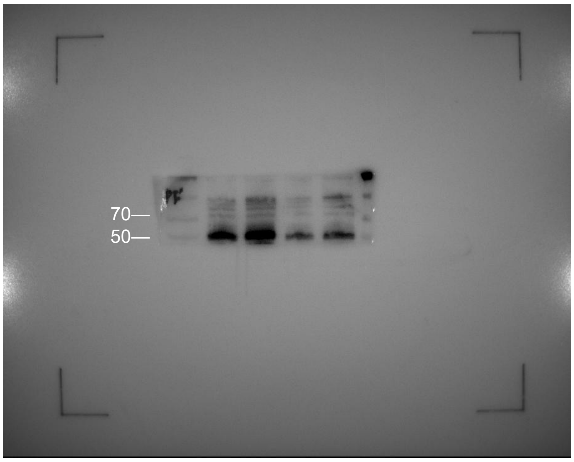

Figure 4E: HEC-1A_p-IRF3

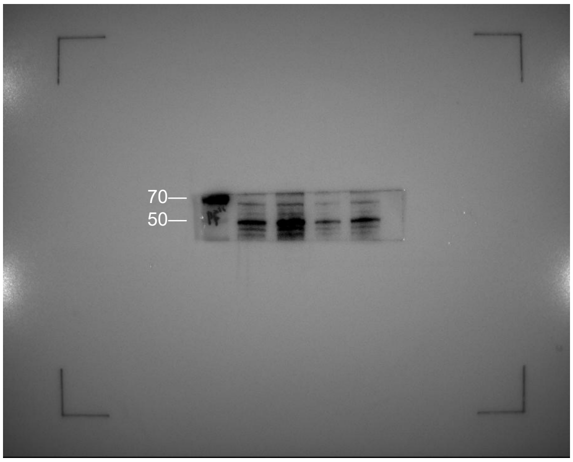

Figure 4E: Ishikawa_GAPDH

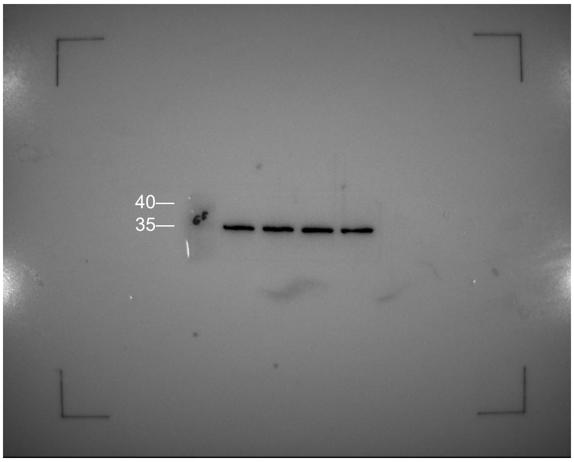

Figure 4E: HEC-1A_GAPDH

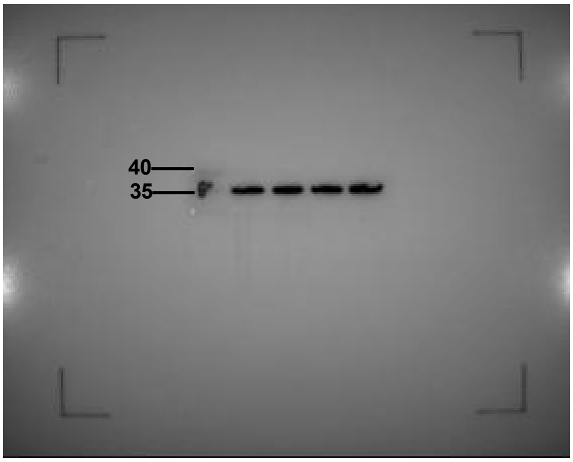

Figure 4F

Figure 4F:Ishikawa_JAML

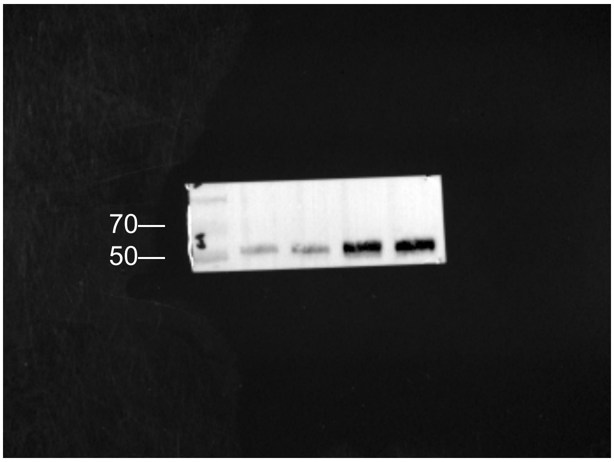

Figure 4F: HEC-1A_JAML

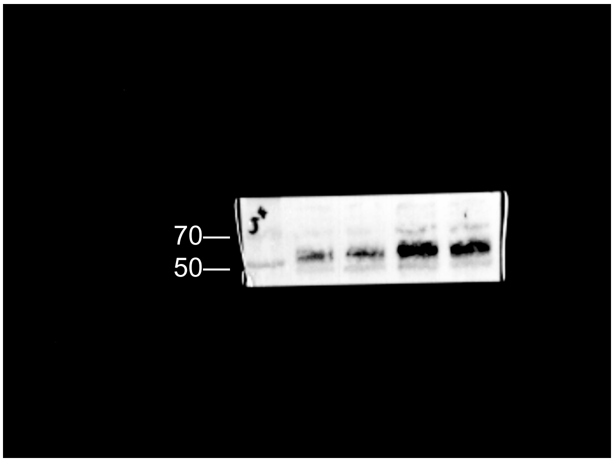

Figure 4F: Ishikawa_cGAS

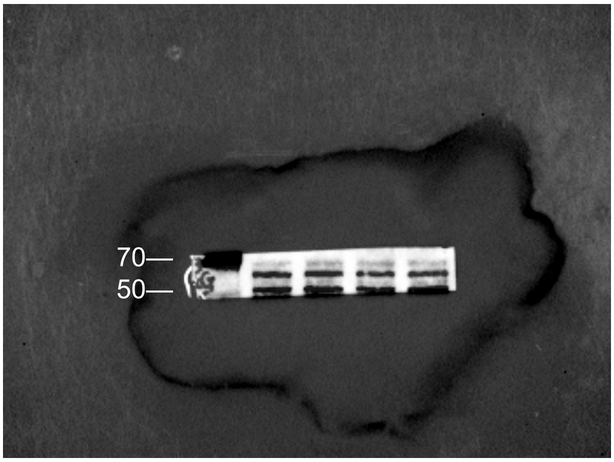

Figure 4F: HEC-1A_cGAS

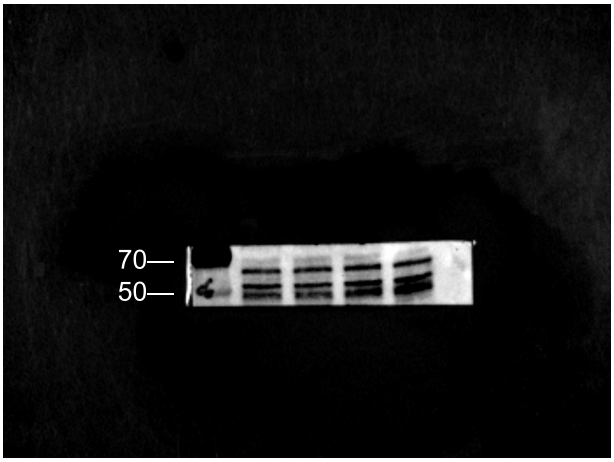

Figure 4F: Ishikawa_STING

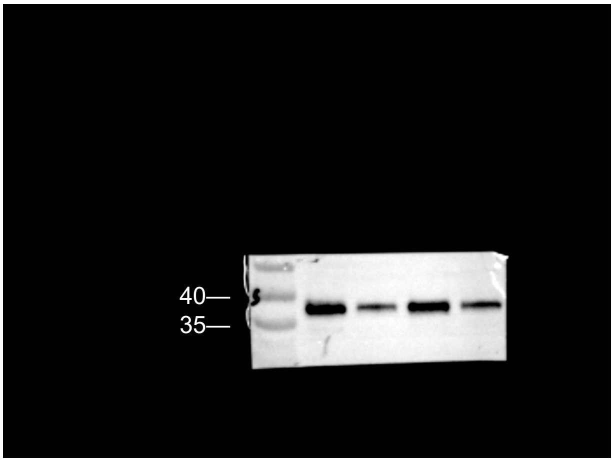

Figure 4F: HEC-1A_STING

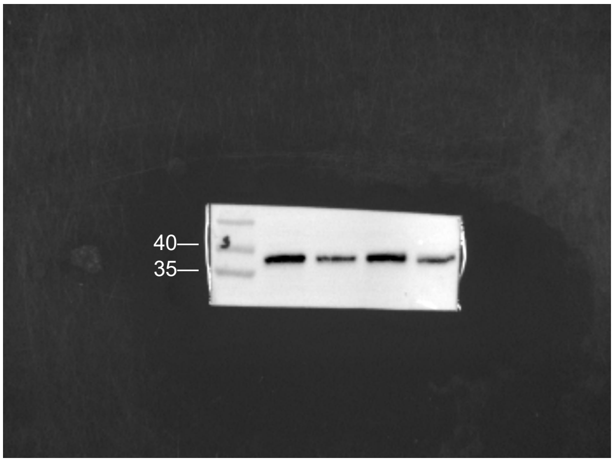

Figure 4F

Figure 4F:Ishikawa_p-STING

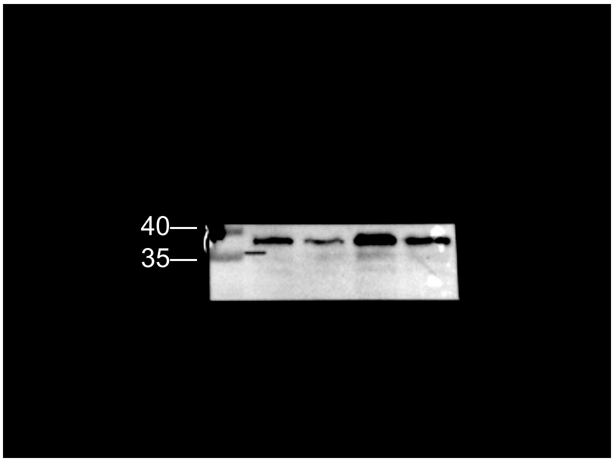

Figure 4F: HEC-1A_p-STING

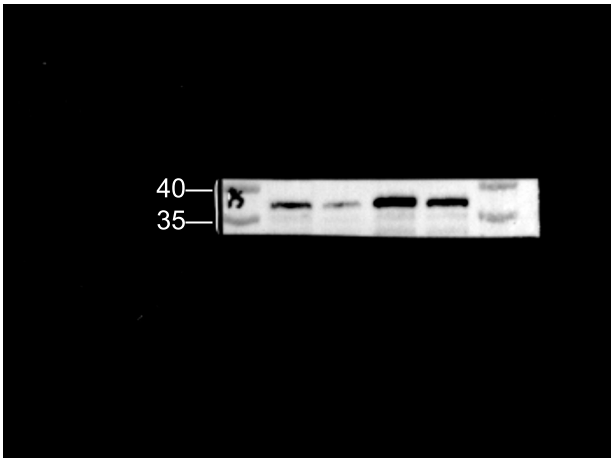

Figure 4F: Ishikawa_TBK1

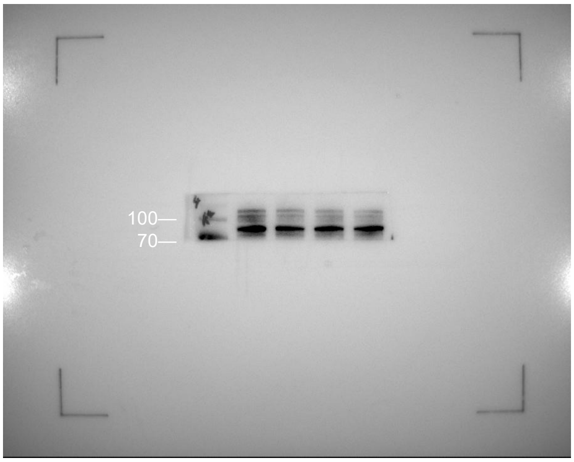

Figure 4F: HEC-1A_TBK1

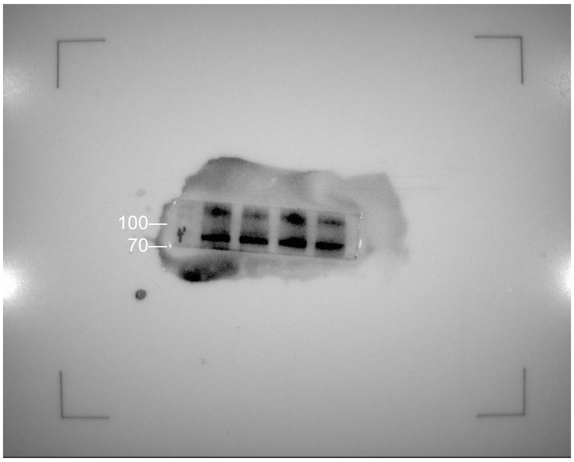

Figure 4F: Ishikawa_p-TBK1

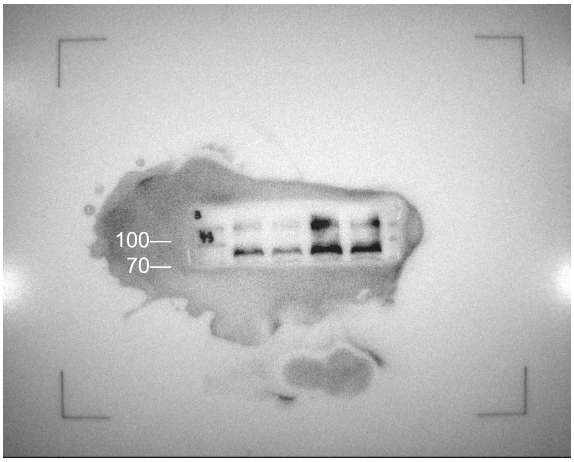

Figure 4F: HEC-1A_p-TBK1

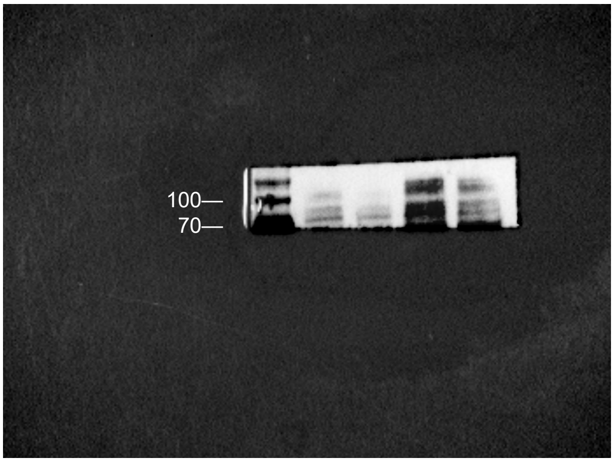

Figure 4F

Figure 4F:Ishikawa_IRF3

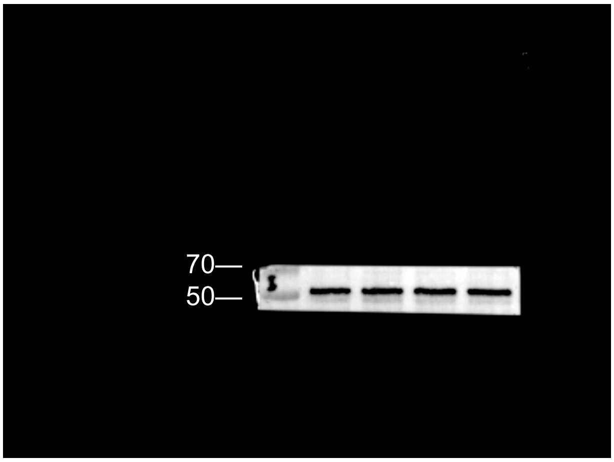

Figure 4F: HEC-1A_IRF3

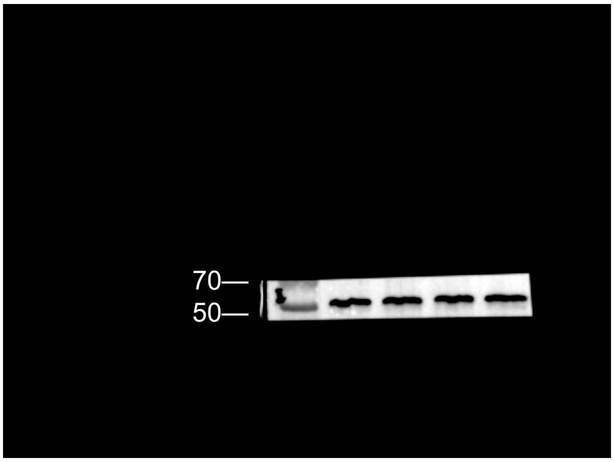

Figure 4F:Ishikawa_p-IRF3

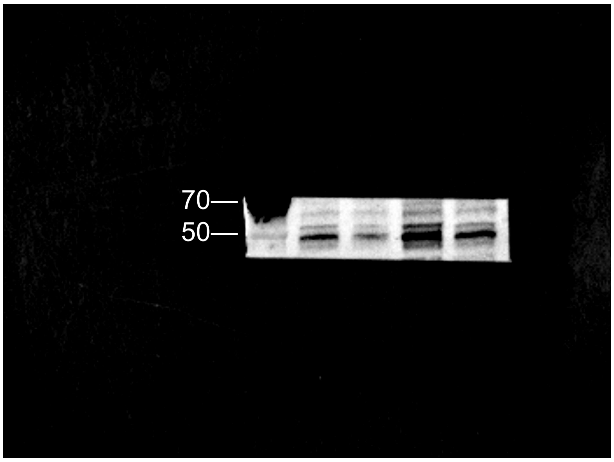

Figure 4F: HEC-1A_p-IRF3

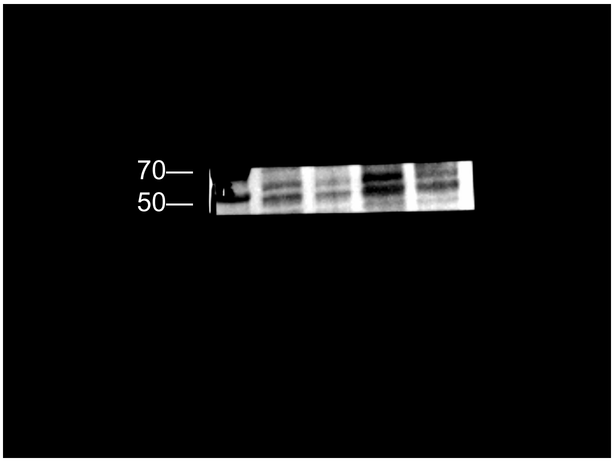

Figure 4F:Ishikawa_GAPDH

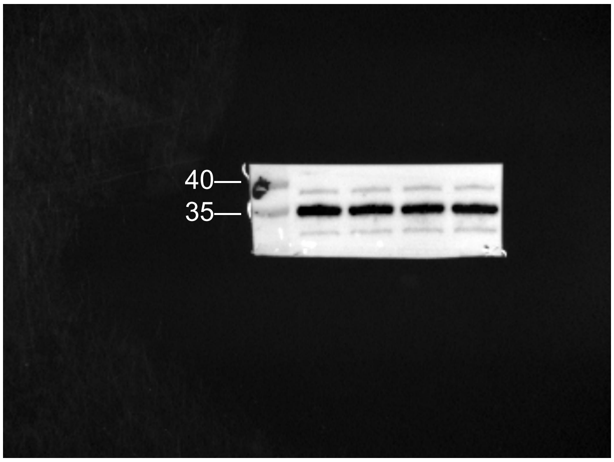

Figure 4F: HEC-1A_GAPDH

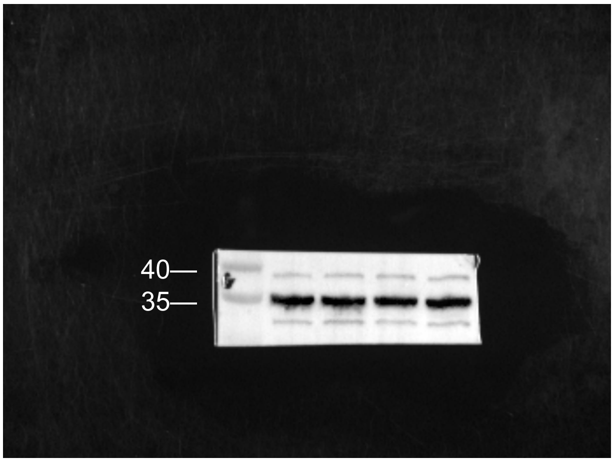

Supplement: Supplementary file 3 [file DataSheet1.pdf]
